# Review of CMOS Integrated Circuit Technologies for High-Speed Photo-Detection

**DOI:** 10.3390/s17091962

**Published:** 2017-08-25

**Authors:** Gyu-Seob Jeong, Woorham Bae, Deog-Kyoon Jeong

**Affiliations:** 1 Department of Electrical and Computer Engineering and Inter-University Semiconductor Research Center, Seoul National University, Seoul 08826, Korea; gsjeong@isdl.snu.ac.kr; 2 Department of Electrical Engineering and Computer Sciences, University of California at Berkeley, Berkeley, CA 94720, USA; wrbae@eecs.berkeley.edu

**Keywords:** CMOS, integrated circuit, photodetector, silicon photonics, transimpedance amplifier

## Abstract

The bandwidth requirement of wireline communications has increased exponentially because of the ever-increasing demand for data centers and high-performance computing systems. However, it becomes difficult to satisfy the requirement with legacy electrical links which suffer from frequency-dependent losses due to skin effects, dielectric losses, channel reflections, and crosstalk, resulting in a severe bandwidth limitation. In order to overcome this challenge, it is necessary to introduce optical communication technology, which has been mainly used for long-reach communications, such as long-haul networks and metropolitan area networks, to the medium- and short-reach communication systems. However, there still remain important issues to be resolved to facilitate the adoption of the optical technologies. The most critical challenges are the energy efficiency and the cost competitiveness as compared to the legacy copper-based electrical communications. One possible solution is silicon photonics which has long been investigated by a number of research groups. Despite inherent incompatibility of silicon with the photonic world, silicon photonics is promising and is the only solution that can leverage the mature complementary metal-oxide-semiconductor (CMOS) technologies. Silicon photonics can be utilized in not only wireline communications but also countless sensor applications. This paper introduces a brief review of silicon photonics first and subsequently describes the history, overview, and categorization of the CMOS IC technology for high-speed photo-detection without enumerating the complex circuital expressions and terminologies.

## 1. Introduction

**Figure 1:**
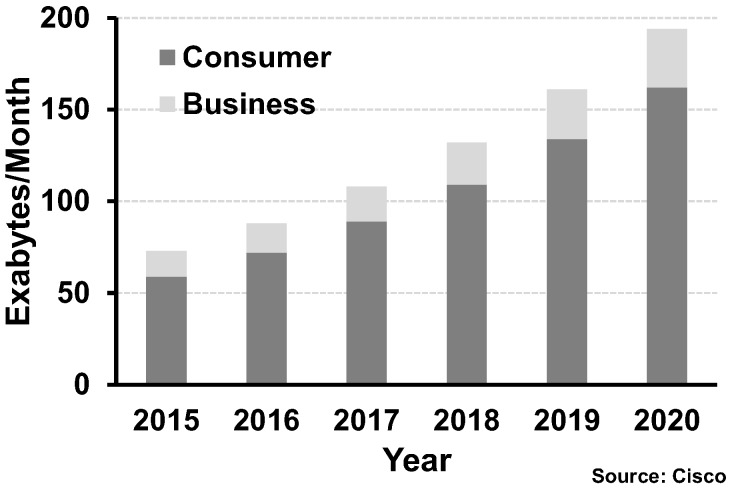
Forecast of total global IP traffic [1].

Today, people’s everyday lives are connected online, i.e., anyone can access or share data worldwide at any time and place. For example, most people today are familiar with accessing streaming media from YouTube, and using social network services and cloud services for personal purposes. The concept of internet of things (IoT) is not new anymore, because IoT devices have already become a part of people’s lives. Two important factors contributing to this revolution are the development of high-performance computing systems and data communication systems which are closely interdependent. Especially, the requirement of high-performance data communication systems is being emphasized more than ever, because of ever-increasing demand for data throughput in every computing system. Even a microprocessor and memory constituting a tiny computing system is now required to handle several hundreds of gigabits per second, and it is growing at a relentless rate. A similar phenomenon is observed in longer-distance applications such as inter-server and long-haul communications. The forecast of data throughput demands is provided by Cisco, one of the leading companies specializing in networking equipment. Figure 1 forecasts the annual global IP traffic by 2020 with a compound annual growth rate of 20%, predicting that the IP traffic would become 200 exabytes/month in 2020 [1].

**Figure 2:**
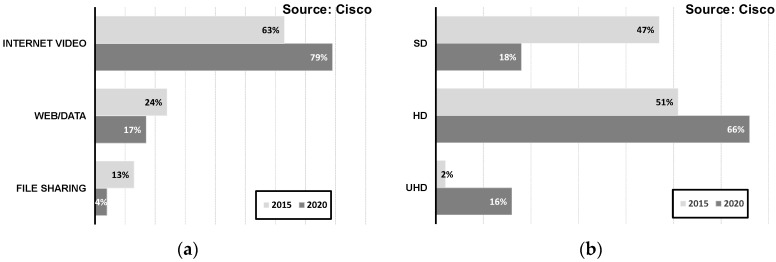
Forecast of global IP traffic (**a**) by application and (**b**) by video content [1].

It is worth investigating the main factors that result in these explosive total data throughput demands. Figure 2a illustrates the breakdown of the total global IP traffic by application. As shown in this figure, the proportion that internet video applications account for will increase in the future. This is reasonably explained in Figure 2b by further subdividing video applications into Standard Definition (SD), High Definition (HD), and Ultra-High Definition (UHD). From these investigations, it is evident that high-quality video contents would be dominant in future IP traffic because of increasing demand for 3D media content and virtual reality. It can also be predicted that the capability of handling high data bandwidth will be required for not only data centers but also end-user devices.

**Figure 3:**
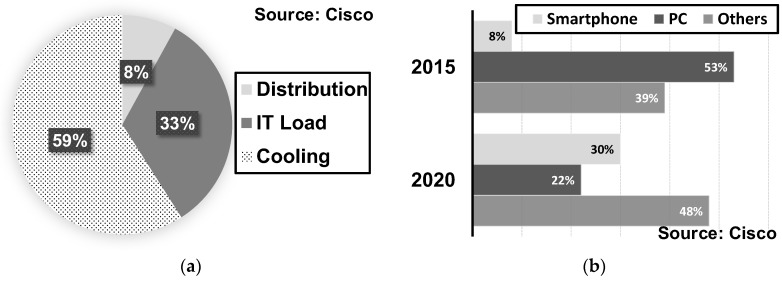
(**a**) Power breakdown of data centers and (**b**) forecast of IP traffic contribution [1].

The above data predicts that the bandwidth requirement will inevitably increase for both data centers and end-user devices. Another important requirement is the reduction of power consumption. Figure 3a shows the power breakdown of typical data centers, indicating that the current communication systems are consuming much power in handling extremely high data rates so that a significant part of resources is being wasted for cooling systems. In the case of end-user devices, the proportion of mobile devices will exceed that of PCs as shown in Figure 3b, meaning the importance of power management will be undoubtedly more pronounced in the future. In summary, it is required to build a reliable communication system satisfying the aforementioned requirements, necessitating innovations in the existing communication systems.

The primary obstacle that hinders high-speed operation is the electrical limitation of the copper-based interconnection which is predominantly used in most applications except for long-reach interconnections. The frequency response of a copper wire severely degrades due to conductor loss and dielectric loss at higher frequencies.

Channel reflection and crosstalk are also detrimental to high-speed operation, limiting the maximum operating frequency to only several GHz over a few tens of meters. Even if various circuit techniques have successfully overcome this physical limit, copper-based interconnection faces the challenge of bandwidth limit as the data rate exceeds several tens of Gb/s. On the other hand, optical interconnection can provide a much higher bandwidth and is free from reflection and crosstalk. In practice, optical fibers have successfully replaced the legacy copper cables over long distances and extension of their usage into shorter distances appears promising. Furthermore, with advanced silicon photonic technologies, it is anticipated that optics can be used even for micro-scale interconnections. Although several problems remain to be solved, many research groups are working toward the same goal, i.e., the realization of high-performance computing and communication systems on a single chip. Silicon photonics with mature complementary metal-oxide-semiconductor (CMOS) IC technologies will provide solutions to fulfill the aforementioned industrial demands. In this paper, we summarize the current status and attempt to forecast the future of optical interconnection in a wide perspective by reviewing papers in as many fields as possible. We also provide a review of key circuit techniques for high-speed photo-detection. This paper is organized as follows. In Section 2, various aspects of optical links are discussed. Subsequently, we focus on the subject of an optical receiver by providing the basis of a photodetector (PD) in Section 3 and the basic topologies of a transimpedance amplifier (TIA) in Section 4. In Section 5 and Section 6, the detailed circuit techniques for the optical receiver are introduced. Finally, we conclude this review work.

## 2. Silicon Photonics for High-Speed Data Communications

### 2.1. Overview of Optical Link

Links or I/O circuits have experienced remarkable advancements and played a major role in the development of data communications systems. However, the bandwidth limitation of copper-based links was already predicted a long time ago and engineers have attempted to determine alternative ways to handling the ever-increasing bandwidth requirement. The optical link based on silicon photonics continues to be the best solution to successfully replace the conventional electrical link [2]. Accordingly, many research groups at not only the leading companies such as Intel and IBM, but also universities and national institutions have focused on developing silicon photonic devices including both the optical and electrical parts [3,4,5]. The results of massive research over several decades appear to be successful and some remarkable results are already applicable to the industrial world [6]. Optical links are gradually replacing their electrical counterparts in long-haul and high-speed applications. Moreover, the ultimate goal of silicon photonics is defined and envisioned as a “macro chip” [7] or an “on-chip server” [8], indicating that the optical links may hopefully substitute most of the electrical links even in the inter-/intra-chip scale. Nevertheless, it appears that the industry is hesitating at adopting silicon photonics as a primary interconnection method for some practical reasons.

**Figure 4:**
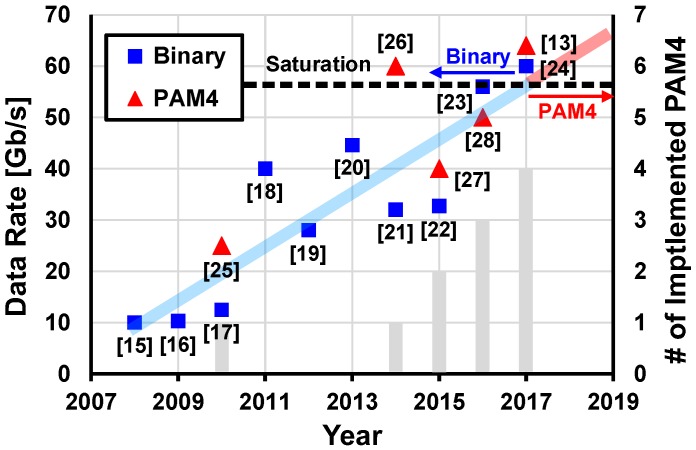
Trends of copper-based electrical links from recent 10-year ISSCC papers.

In the case of very short-reach applications ranging from several centimeters to meters, electrical links are still dominant, despite the bandwidth limit of copper-based interconnection. The proliferation of copper-based links in this area can be mainly attributed to the advancements of CMOS technologies and circuit techniques. By utilizing advanced CMOS technologies and equalization techniques, operations at higher than 25 Gb/s even in severe loss conditions of more than 40 dB were achieved [9,10,11]. Recently, circuit designers attempt to overcome the limitations of copper by introducing a pulse-amplitude-modulation (PAM) signaling that can enhance the effective data rate for the same loss condition as the conventional binary signaling [12,13,14,15]. Figure 4 summarizes the binary and PAM-4 transceivers which recorded the fastest operating speed each year over the past 10 years [14,16,17,18,19,20,21,22,23,24,25,26,27,28,29]. Notably, the electrical links face the challenge of bandwidth limitation and the maximum available speed would saturate in the immediate future. Therefore, designers are focusing on developing PAM-4 transceivers in recent years. However, despite the innovations in electrical links, one can expect that the capacity of the copper-based links would saturate eventually because of both the circuit and device limitations.

**Figure 5:**
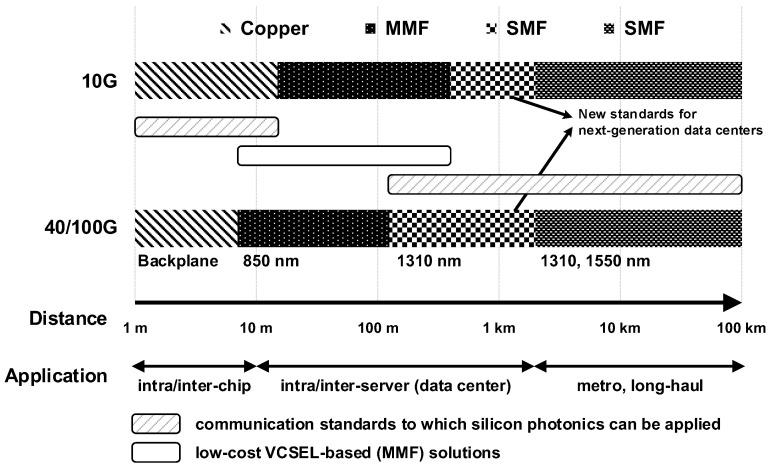
Summary of communication standards, Ethernet.

The optical links can be configured with either a multi-mode fiber (MMF) or a single-mode fiber (SMF). An MMF-based link features a distinctly low cost whereas the communication distance is strictly restricted due to modal dispersion. A vertical-cavity surface-emitting laser (VCSEL) is commonly used as a light source in the MMF-based links with a typical wavelength of 850 nm. Owing to the low cost of MMF-based links, short-reach applications ranging up to several hundreds of meters mostly rely on them. On the other hand, an SMF-based link is free from modal dispersion, thereby providing a much longer communication distance and is only limited by chromatic dispersion and other loss mechanisms such as Rayleigh scattering or electronic absorption. Typical communication standards based on SMF support a communication distance of up to several tens of kilometers with wavelengths of 1310 or 1550 nm. Figure 5 summarizes the communication trends from Ethernet standards: 10 GbE, 40 GbE, and 100 GbE [30,31]. Among various optical communication applications, the short-reach applications are the most popular and market size is growing rapidly owing to increasing demand in data centers and active optical cables (AOCs). Especially, the demand for the AOCs would increase significantly, as their usage is not restricted to data centers, but massively extended to everyday life. However, since silicon photonics is based on a silicon waveguide, only the telecom wavelengths are available in silicon photonics, which is not directly compatible with the short-reach applications, most of which rely on low-cost VCSEL-based solutions. Nevertheless, the prospect of silicon photonics is promising for the future inter-/intra-chip interconnections and the next-generation data centers covering a communication distance of up to 2 km as highlighted in Figure 5 [32,33].

### 2.2. Silicon Photonics

In this section, an overview of silicon photonics is presented and silicon photonic technologies are discussed with some examples of silicon photonic transceivers. The current status of silicon photonics and the underlying problems to be solved in the future are addressed.

As explained in [34,35], outstanding progress in silicon photonics was first observed in the early 2000s. Although silicon photonics pursues the realization of on-chip optical interconnections, they may find immediate application in fiber-optic communications as discussed in the previous section. Therefore, extensive research on silicon photonics include not only lasers, photodiodes, modulators, and waveguides but also waveguide-to-fiber couplers. Furthermore, all the photonic devices necessary to build a complete photonic transceiver were already viable in 2000s except for the laser source. A more recent review work states that the implementation of silicon lasers is still challenging; however, a number of innovations using bonding and epitaxial growth of III-V materials on silicon have been accomplished, so called a hybrid silicon laser [36]. In the case of photodetectors, silicon by itself cannot be a good absorber for telecommunication wavelengths due to its large bandgap energy. Among the numerous silicon-based photodiodes, a germanium-introduced photodiode is the most popular photodetector; moreover, recent germanium photodiodes have achieved very high bandwidths of several tens of GHz [37,38]. Silicon-based photodiodes will be discussed again in the following section. Silicon modulators are typically based on plasma dispersion effect that the refractive index of the waveguide is changed by the density of free carriers, thereby enabling the amplitude modulation with Mach-Zehnder interferometer (MZI) structures. Modern silicon MZI modulators are capable of handling data rates higher than 50 Gb/s [39,40]. Another type of silicon modulators is a ring resonator which exhibits a small footprint and better energy efficiency than the MZI structures [41,42]. Furthermore, a wavelength-division-multiplexing (WDM) is readily applicable to the ring-resonator-based architectures, which is a desirable feature for future silicon photonic transceivers [43]. Much progress has also been made in silicon waveguides and couplers. Depending on the type of the waveguide, typical propagation loss can be several dB/cm or less than 1 dB/cm [36]. A coupling technique is also of great importance for successfully interfacing with an off-chip optical fiber. Fortunately, a number of good solutions such as holographic lens have been proposed so far, ideally exhibiting an extremely low-loss condition which is comparable to that of the conventional fiber connector [6].

Despite the brilliant success of silicon photonic ICs (PICs), compatibility with electronic ICs (EICs) or a monolithic integration of the PICs and the EICs is still challenging. Despite many efforts to realize electronic and photonic ICs (EPICs) on a bulk CMOS platform, the overall performance has yet to be improved. Alternatively, an SOI CMOS platform is generally accepted as an ideal platform for the implementation of both silicon waveguides and active transistors.

**Figure 6:**
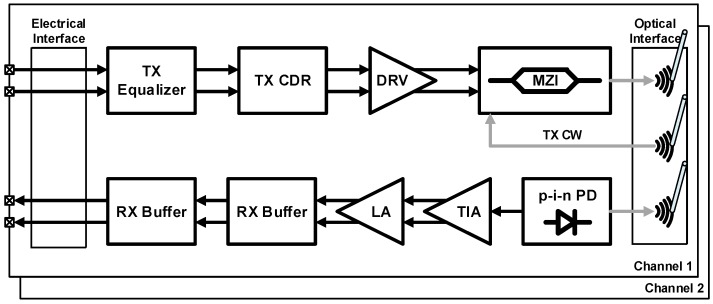
2 × 10-Gb/s dual channel optoelectronic transceiver [44].

In a standard 0.13-μm CMOS silicon-on-insulator (SOI) platform, the first fully integrated optoelectronic transceiver was realized by Luxtera in 2006 [44]. The implemented chip achieves a total throughput of 20 Gb/s with a dual-channel architecture using an SMF for long-reach communication. The overall block diagram of the transceiver is illustrated in Figure 6. The TX converts an electrical signal into a data-modulated optical signal and the RX operates in the other way around. The TX equalizer in the front compensates for the frequency loss of the electrical channel prior to the TX input. Following the equalizer, the TX-side clock and data recovery (CDR) retimes the data to reduce jitter and produce a clean data output. The driver further increases the amplitude of the data signal to make an optimum input condition for the MZI. The MZI is based on a reverse-biased p-n junction for high-speed operation at the cost of reduced phase shift, thus reducing the extinction ratio (ER). ER can be raised by increasing the length of the modulator or enlarging the driver output swing. The driver output swing and the length of the modulator in this design are 5 V_ppd_ and 4 mm, respectively. A holographic lens is used as a fiber-optic coupler for both the TX and the RX. The PD at the RX is flip-chip bonded to the silicon die. The transimpedance amplifier (TIA) converts the PD current to a voltage which is further amplified by a limiting amplifier (LA) for the proper operation of the CDR. Following the CDR, an output buffer is employed to drive a differential 100-Ω transmission line. In 2007, Luxtera further developed their photonic device library by adding a dense WDM (DWDM) building block. By implementing DWDM multiplexers and demultiplexers, they succeeded in integrating a 4 × 10-Gb/s DWDM optoelectronic transceiver in a 0.13-μm CMOS SOI technology [45].

**Figure 7:**
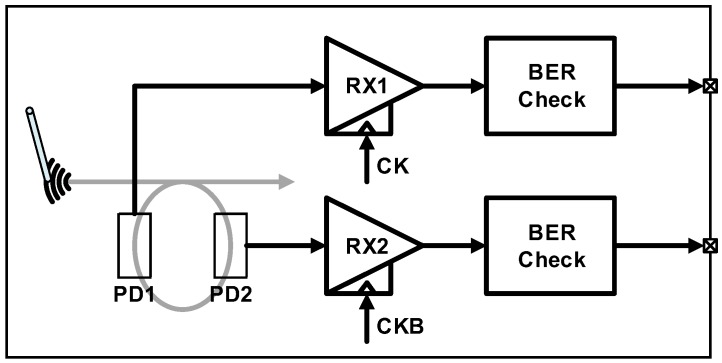
Monolithic receiver with PD splitting [46].

The silicon photonics research group at MIT has also published successful works in monolithic optical transceivers. In 2012, the first monolithic optical receiver in a sub-100-nm standard SOI process was implemented [46]. In this work, novel approaches are proposed toward implementing a PD and a detecting scheme, which can be attributed to the increased design flexibility of the PD in the monolithic integration platform. An important advantage of the monolithic integration is that low PD and wiring capacitances can be obtained. An integrating receiver illustrated in Figure 7 can be the most appropriate topology by exploiting the low-capacitance condition and the presence of an RX clock. However, an insufficient timing margin for evaluation can severely degrade the sensitivity. PD splitting combined with a double-data-rate (DDR) scheme is implemented in this work to alleviate the timing margin. By interdigitating the metal contacts of the PD, it can be divided into two separate PDs sharing a common waveguide, thus reducing the photocurrent of each path by half. In spite of the halved photocurrent, the PD splitting is more advantageous at higher data rates considering that the sensitivity degrades exponentially with the shortened evaluation time. Another improvement is at the PD structure that incorporates a ring resonator to confine the light, thereby enhancing the absorption with a smaller PD length. The implemented receiver chip operates at the data rate of up to 3.5 Gb/s with an extremely high energy efficiency of 50 fJ/b. This demonstration is significant in that it paved the way for future memory-processor interfaces based on silicon photonics.

A monolithic optical transceiver operating at higher speed was also reported jointly by UC San Diego and Oracle in the same year [47]. The chip is implemented in a 0.13-μm SOI CMOS technology and achieves an operating speed of 25 Gb/s with an energy efficiency of 10.2 pJ/b for the entire transceiver. The optical TX is based on a micro-ring modulator and the optical RX employs a Ge photodetector. Notably, an asymmetric pre-emphasis is used even for the reverse-biased ring modulator. When the optical devices are forward-biased, or more specifically when operating a VCSEL or a ring modulator in a forward-biased condition, the storage time of minority carriers obstructs the operating speed and causes asymmetric rising and falling times. On the other hand, in the case of a reverse-biased ring modulator, the photon lifetime dominates the maximum operating speed, which also incurs asymmetry. Therefore, the asymmetric pre-emphasis, or independently controlling the rising and falling edges, is essential even for the reverse-biased condition in order to improve the signal quality at higher speed. 

**Figure 8:**
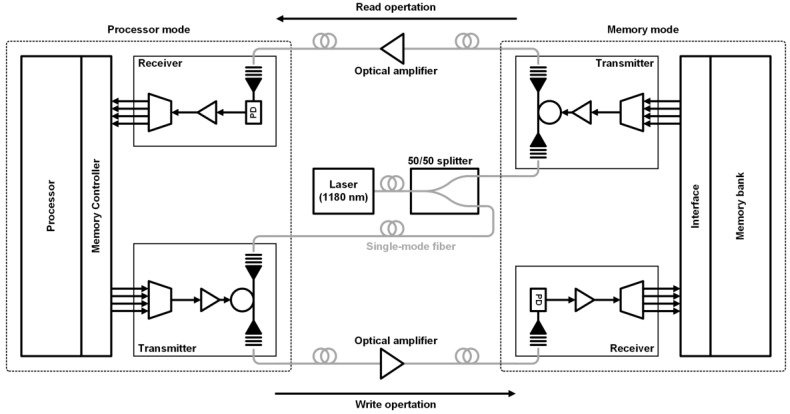
Block diagram of memory-processor optical link realized in [48].

In 2015, a more sophisticated work was performed by collaboration among several research groups in academia and industry [48]. They demonstrate good feasibility of future processor-memory links by implementing a processor, a memory, interface circuits, and photonic devices altogether onto a single microchip in a commercial 45-nm CMOS SOI process without any changes to the foundry process as shown in Figure 8. The implemented chip reliably integrates 70 million transistors and 850 photonic components. The functionality of the processor-memory link is also verified by demonstrating actual read and write operations between the processor and the memory at 2.5 Gb/s. Although only a single-wavelength operation is demonstrated, the total aggregate bandwidth can be increased by more than 10 times without using additional fibers. A single external laser source is employed to supply 1180-nm continuous wave for both processor and memory sides using the 50/50 power splitter. The receiver incorporating a SiGe PD exhibits a sensitivity of −5 dBm for the bit-error rate (BER) of 10^−12^. At the transmitter, a micro-ring resonator with reverse bias is employed and showed an ER of 6 dB. Since the resonant wavelength of the ring structure is sensitive to not only physical dimension but also temperature, a continuous stabilization of the wavelength is essential. In this work, a small part of the modulator output power is monitored and the resonant wavelength is tuned in a way that maximizes the output power by digitally controlling an embedded resistive heater inside the ring. Despite several limitations, the contribution of this work is evident and further development is expected to realize more practical silicon photonic systems in the near future.

On the other hand, the bulk CMOS platform has been relatively less popular than the SOI counterpart primarily due to the difficulty of implementing low-loss silicon waveguides. However, the realization of silicon photonics in bulk CMOS would be preferred because the bulk CMOS technology has been the mainstream so that most electronic parts are optimized in bulk CMOS processes with the lowest cost. On the other hand, the floating-body effect of SOI CMOS may lead to non-linear characteristics of the active transistors and heat dissipation can be problematic, thus rendering the SOI platform inadequate for the extremely high level of integration. Therefore, photonic systems on the bulk CMOS platform have also gathered significant attention.

**Figure 9:**
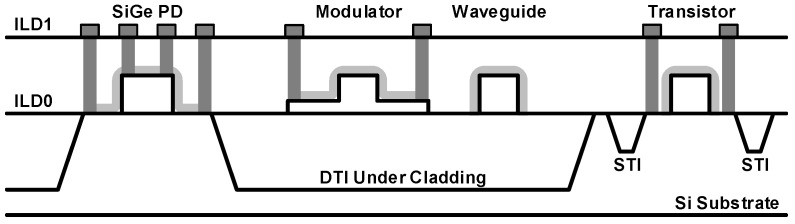
Cross-section of photonic and electronic devices [49].

In 2014, the first monolithic integration of photonic and electronic devices on the bulk CMOS was reported [49]. The most challenging part is the waveguide integration with a low propagation loss. Figure 9 shows the cross-section of the implemented silicon photonic devices. For the formation of a polysilicon-based waveguide, a lower cladding and an upper cladding are implemented using deep trench isolation (DTI) and silicon nitride barrier films between the inter-level dielectric (ILD) layers, respectively. Several optimization processes such as the crystallization of amorphous polysilicon and low-pressure chemical vapor deposition (LPCVD) of silicon nitride are also performed to further reduce the loss of the waveguide. The resulting waveguide achieves a record attenuation of 10.5 dB/cm which is sufficiently low to support resonant ring modulators. A polysilicon resonant detector and a SiGe p-i-n detector are both implemented. The fully functional optical transceiver operates at 5 Gb/s exhibiting an energy efficiency of 2.8 pJ/b.

**Figure 10:**
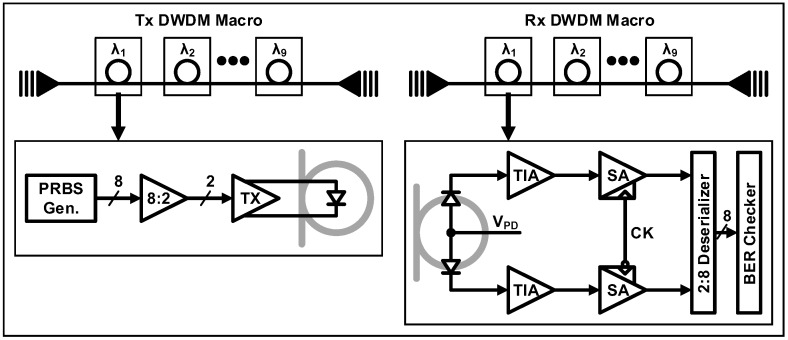
Monolithically integrated DWDM optical link in bulk CMOS [50].

Subsequently, a more advanced work was demonstrated by incorporating a DWDM system into the bulk CMOS platform [50]. A DWDM optical transceiver is monolithically integrated on a 0.18-μm bulk CMOS process with all the optical devices implemented using polysilicon without relying on the epitaxial crystallization of silicon or the introduction of Ge. By applying the same approaches presented in [49], low-loss photonic devices including waveguides, couplers, micro-ring modulators, and PDs can be successfully integrated with minimal modifications to the original CMOS process. Moreover, based on the micro-ring structures, 9-wavelength TX and RX DWDM macros are also realized as shown in Figure 10. The TX employs an internal pseudorandom binary sequence generator, a serializer, and a modulator driver, enabling electro-optic conversion by the carrier-depletion micro-ring modulator. At the RX side, a polysilicon-based PD utilizing absorption from defect states, combined with the resonant structure, provides an acceptable responsivity of 0.2 A/W. Similarly, the PD splitting technique in [46] is also employed to mitigate the timing margin. After the DDR-based receiver front-end, the data is further deserialized for the estimation of BER. In order to achieve a stable wavelength locking of the DWDM system, a heater and a digital logic controlling the heater are also implemented. Consequently, for a total aggregate data rate of 45 Gb/s, a 9-wavelength DWDM link sharing a single bus waveguide is successfully demonstrated.

**Figure 11:**
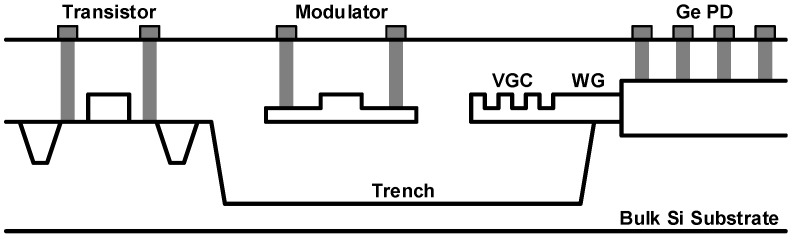
Schematic view of EPIC structure [51].

Silicon photonics based on bulk-Si substrate is also an attractive option for processor-memory interfaces, especially for cost-sensitive dynamic random-access memory (DRAM) applications [51]. Processor-memory interfaces are currently facing two challenging industrial requirements. The first requirement is the demand for higher bandwidth, according to which the next-generation DRAM (DDR5) standard requires the maximum per-pin data rate of 6.4 Gb/s. The other requirement is that the total memory capacity that a memory controller can handle should be maximized. However, the existing schemes, such as the multi-drop bus topology or the point-to-point bus topology, cannot satisfy the aforementioned requirements simultaneously. Fortunately, if an optical interconnection is applied to the multi-drop bus interface, the per-pin data rate can be increased significantly without degrading the total available memory capacity. The CMOS SOI platform can never be adopted in the DRAM interfaces where cost is a crucial factor. Samsung has presented several works based on the bulk-Si platform, which is different from the platform in [50] mainly in terms of waveguide-fabrication techniques. The schematic view of integrated EPICs is shown in Figure 11. Unlike in [50], the waveguides are formed through a local crystallization based on epitaxy. For that purpose, a trench is formed first and filled with silicon dioxide. Subsequently, amorphous silicon (a-Si) is deposited using LPCVD. After the deposition, a-Si is crystallized by solid-phase epitaxy (SPE) using the bulk-Si substrate near the edges of the trench as a crystal seed, thereby growing the crystal laterally toward the center of the trench. Finally, the waveguide is patterned using dry etching. Owing to the introduction of epitaxy, a higher quality of crystallization as compared with the previous works is obtained, exhibiting a waveguide loss of only 3 dB/cm. A further improvement can be achieved using a laser-induced epitaxial growth based on the liquid phase epitaxy method, resulting in an almost perfect crystallization, so that the grain size is comparable to that of the bulk Si. Using the waveguides formed by the SPE method, both the MZI and the micro-ring modulators are realized. Two types of PDs are also implemented by introducing Ge on Si: a surface-illumination type and a butt-coupled-waveguide type. Although the photonic devices alone exhibited good performance, overall performance degradation is observed when integrating the EIC and the PIC together with the combined process, which needs to be improved through process optimizations. 

**Figure 12:**
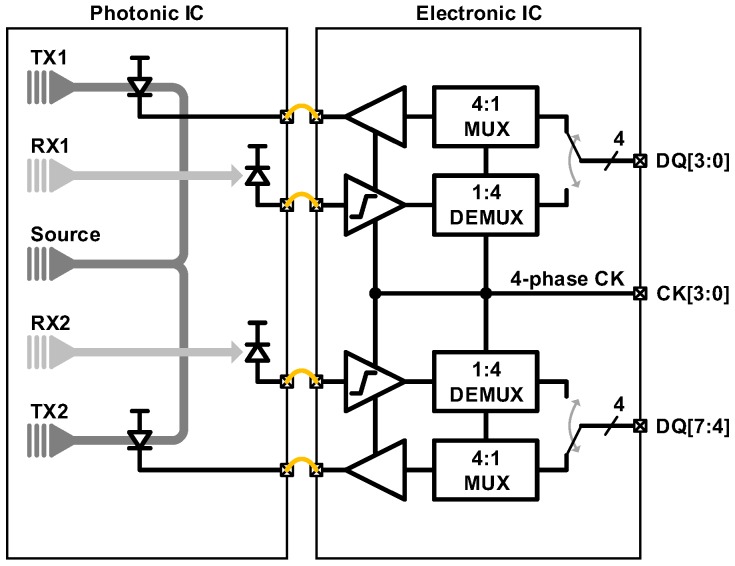
Block diagram of optical transceiver [51].

Samsung has also demonstrated the feasibility of a multi-drop bus topology based on silicon photonics. Figure 12 illustrates an optical link with the hybrid integration of the EIC and the PIC, which shall be developed into a fully monolithic EPIC in the future. The implemented optical link consists of two sets of TXs and RXs processing 8 DQ signals, and simply emulating a read or a write operation of the controller-memory communication. The link successfully demonstrates an error-free operation at the data rate of up to 2.5 Gb/s per fiber.

### 2.3. Hybrid Integration

As briefly discussed so far, silicon photonics has undergone remarkable progress, demonstrating its potential to replace the legacy copper-based interconnections. Nevertheless, some issues have yet to be addressed to prevent degradation of the performance of the EPIC. Moreover, the compatibility with a FinFET CMOS platform is unknown and should be investigated in the future. Alternatively, a hybrid integration leveraging the respective process optimizations of the EIC and the PIC would be more advantageous at present, and it has become viable owing to advanced 3-D integration techniques. In this section, several recent works based on the hybrid integration will be reviewed and compared with the monolithic integration in various aspects.

**Figure 13:**
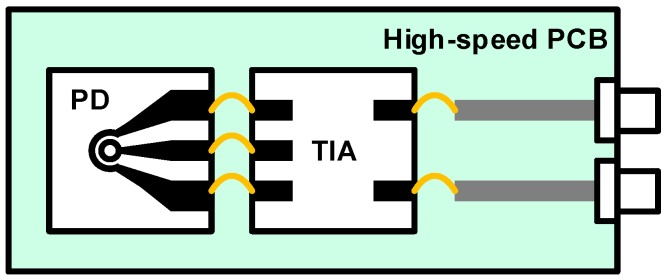
Illustration of wire-bonded EIC and PIC.

The most common integration method is the use of a wire-bonding technique which has been prevalently used in EIC packages. However, wire bonding severely degrades the signal integrity as the data rate becomes higher, because the length of a wire is directly translated into an inductance. For even higher data rates, the situation degrades as the wavelength of the signal becomes comparable to the length of the wire. Due to its relatively large dimension, wire bonding also limits the maximum pin density; hence, it is not appropriate for modern system-on-chip packages where an extremely high pin density is required. Nevertheless, thanks to its distinguishably low-cost characteristic, it is still widely used for low-speed packages and chip-on-board test environments. An example of the integration of EPIC based on wire bonding is shown in Figure 13. A typical PD can be wire-bonded to a TIA chip and the output of the TIA is connected to a trace on a printed circuit board (PCB) to interface with high-speed end-launch connectors. Based on wire bonding, the works in [52,53] achieve operating data rates of 25 Gb/s and 64 Gb/s, respectively. 

**Figure 14:**
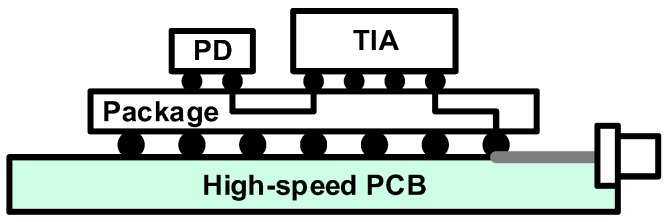
Illustration of flip-chip package [54].

Flip-chip bonding is a more advanced technique based on face-to-face bonding, completely eliminating the bond wires. Because of a shortened chip-to-chip space (several tens of μm with modern techniques), the parasitic elements can be significantly reduced, resulting in improved signal integrity at higher speeds. Figure 14 illustrates a typical flip-chip package. The PD chip and the TIA chip are flip-chip bonded on the same package substrate through which high-speed signal interconnection is made. The output of the TIA is connected to the PCB trace via the package and the solder bump. In this case, the structure of the PICs should be a back-illumination type for proper fiber coupling. With this configuration, the work in [54] presents a 4 × 28-Gb/s optical receiver exhibiting good sensitivity. 

**Figure 15:**
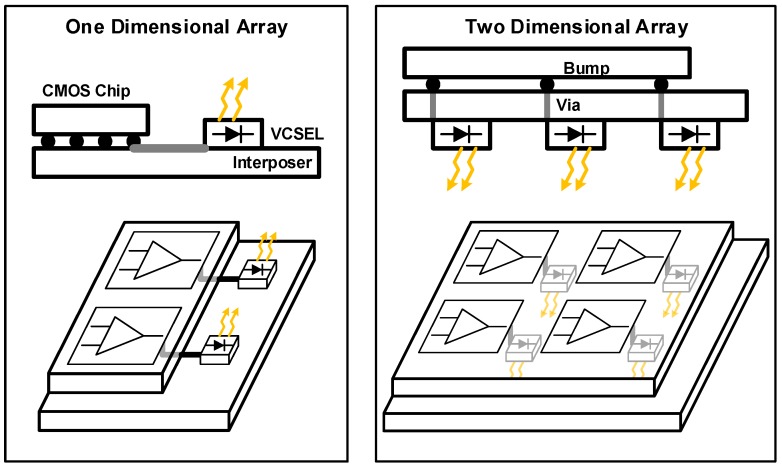
Implementation of 2-D array of EPIC [55].

Similarly, but in a slightly different manner, the EIC and the PIC are stacked vertically using an interposer as shown in Figure 15 [55], realizing a 12 × 5 two-dimensional EPIC array in order to maximize the I/O density. The implemented transceiver chip achieves a total aggregate data rate as high as 600 Gb/s.

**Figure 16:**
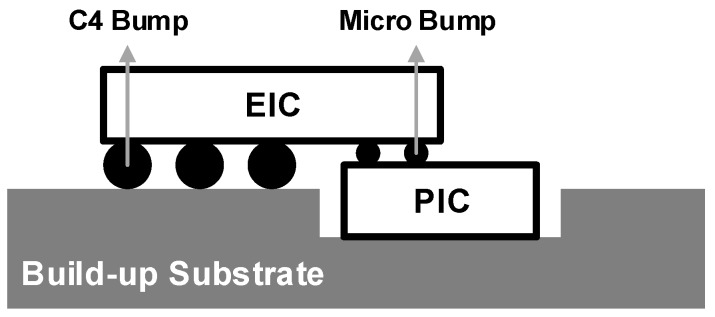
Flip-chip bonded EIC directly on PIC using micro bump [56].

Figure 16 illustrates an improved flip-chip package that directly bonds the EIC and the PIC using micro bumps, thus obviating any redistribution layer between the high-speed regions to further reduce the parasitic components. The rest of the signals are connected to the package substrate through C4 bumps. An additional cost may be incurred by such a package technique due to the requirement of a customized substrate.

**Figure 17:**
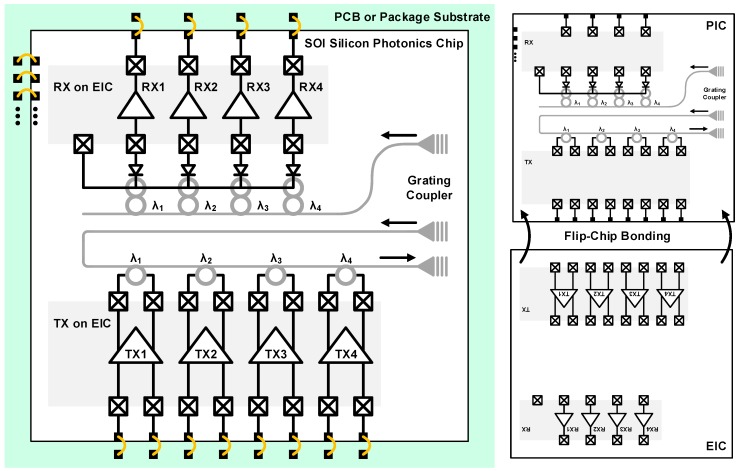
EIC flip-chip bonded to macro-PIC [43].

Currently, the most advanced technique is shown in Figure 17. Contrary to the previous technique, it uses the PIC as a redistribution layer itself, thus simplifying the package process [40,43,57]. The EIC is flip-chip bonded to the macro PIC in a similar way. Not only the high-speed signal pads but also the low-speed signal pads are connected to the PIC. Subsequently, the low-speed signals are carried outside the PIC through a typical wire-bonding package. Based on this technique, the work in [43] demonstrates a 4 × 20-Gb/s WDM transceiver and [40] implements a 56-Gb/s optical transmitter with the MZI structure.

In Table 1, we summarize and compare the optical transceivers presented so far. Notably, the overall performance of the hybrid-integrated transceivers is better than that of the monolithic transceivers, especially in terms of energy efficiency. This can be attributed to the independently optimized processes of the EIC and the PIC. However, it should also be noted that recent monolithic transceivers have demonstrated significant improvement, and are comparable to their hybrid counterparts.

## 3. Si-Based Photodetectors

In this section, we examine the basic terminologies of a PD. We also briefly discuss several PD structures based on silicon photonics and their operating principles.

### 3.1. Basic Terminology

We first present some terminologies related to the PD characteristics. The quantum efficiency is the ratio of the number of generated electron-hole pairs to the number of incident photons. Since the quantum efficiency directly determines the sensitivity of the entire receiver, designing a PD to have a high quantum efficiency is crucial. This quality is generally expressed as:

(1) $$\eta =\frac{I_{ph}/q}{P/h\nu }$$

The internal quantum efficiency is sometimes defined by de-embedding the loss occurring at the detector interface to evaluate the detector performance alone. The responsivity is a more frequently used quantity by engineers with a similar meaning to the quantum efficiency, and can be expressed as:

(2) $$R=\frac{I_{ph}}{P}=\frac{\eta q}{h\nu }=\eta \frac{\lambda \left(nm\right)}{1240}$$

The bandwidth of the PD is determined by two time constants: the transit time and the RC time constant which arises from the series resistance and the junction capacitance of the PD. Hence, the total time constant should be considered for the estimation of the bandwidth as follows:

(3) $$\tau =\sqrt{\tau_{transit-time}^{2}+\tau_{RC}^{2}}$$

### 3.2. Si PD

**Figure 18:**
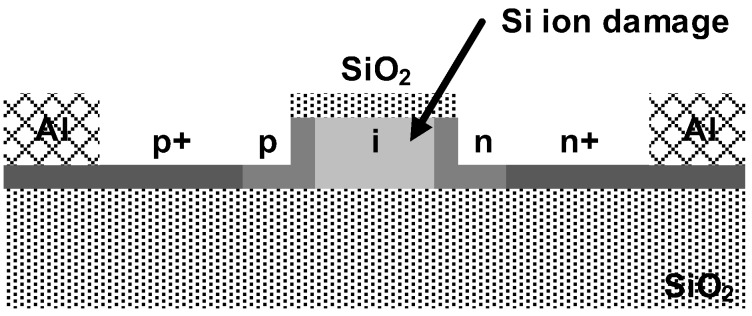
Cross-sectional view of waveguide PD [58].

Silicon is a good waveguide material for telecommunication wavelengths due to its large bandgap energy equivalent to the energy of light with the wavelength of approximately 1100 nm. Therefore, ironically, silicon cannot be a good absorber for wavelengths longer than 1100 nm. Fortunately, some promising results have been obtained by enabling a sub-bandgap detection without introducing any other materials. It is known that silicon can also detect light of wavelength longer than 1100 nm if crystal defects are present, which cannot be easily explained theoretically, but can be proven experimentally [58,59,60,61,62]. By deliberately introducing defects into a p-n diode in a waveguide structure, the sub-bandgap detection in silicon can be realized. In [58], a silicon waveguide PD is implemented, wherein the quantum efficiency is enhanced by applying ion implantation. The implemented waveguide PD is illustrated in Figure 18. The diode is formed on a SOI substrate using a standard CMOS process. After the formation of the waveguide PD, the ion implantation is conducted to introduce the crystal defects. The lengths of the waveguides are 0.25 and 3 mm. The PD with a waveguide of length 0.25 mm shows a responsivity of sub-0.1 A/W at low reverse voltages; however, the responsivity can approach 1 A/W by increasing the reverse voltage at the cost of increased dark current, thereby degrading the minimum detectable power (MDP). The PD with a waveguide of length 3 mm can absorb virtually all the light, thus enhancing the responsivity at lower reverse voltages. However, the increased length directly results in higher junction capacitances, which severely degrades the frequency response. The estimated bandwidths of the PDs with the waveguides of lengths 0.25 and 3 mm are 10–20 GHz and 2 GHz, respectively. Further research advances the performance of the silicon waveguide PD through several process optimizations, providing a bandwidth of >35 GHz and an internal quantum efficiency of 0.5 to 10 A/W [59].

**Figure 19:**
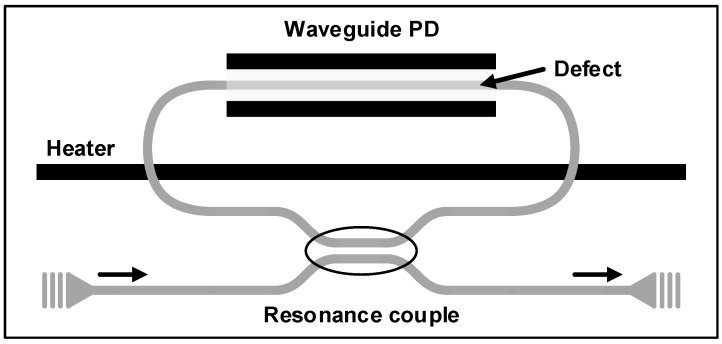
Schematic view of silicon resonator-enhanced PD [60].

As clearly observed in [58,59], the silicon waveguide PD suffers from the tradeoff between the quantum efficiency and the bandwidth. In order to overcome this severe tradeoff, the work in [60] suggests that the adoption of a ring resonator can enhance the absorption with significantly reduced device dimension. As shown in Figure 19, the resonator-enhanced PD achieves a responsivity of 0.14 A/W with a length 10 times shorter than that of the straight waveguide for the same responsivity. The dark current level is also maintained below 0.2 nA, thus exhibiting an MDP of only 1.4 nW. Moreover, a resistive heater is employed for tuning the resonance wavelength.

The silicon PDs discussed so far are all based on the SOI platform. However, the realization of a silicon PD on the bulk CMOS platform would be essential for a truly monolithic silicon photonic receiver. In [61], starting with a bulk silicon substrate, a micro-ring resonator PD using polysilicon is demonstrated, achieving a responsivity of 0.15 A/W, a dark current level of 40 nA, and a gigahertz frequency response. Recently, the work in [62] achieves further improvements of overall performance by applying mid-level implants to a typical p-i-n structure. The recent advances in silicon PDs are significant because they simplify the overall process with a minimal addition of steps, indicating a better compatibility with the standard CMOS processes than their Ge-based counterparts. Especially, the silicon PD incorporated in a waveguide has a distinct advantage that the integration with a waveguide is readily realized, which is challenging for the Ge-based detectors. Furthermore, the resonator-based structure directly enables the WDM function, which is a desirable feature for highly dense interconnection [50].

### 3.3. Ge-Introduced PD

Another approach involves the introduction of Ge which has a smaller bandgap energy than Si. By appropriately combining Si with Ge, the detectable wavelength can be extended from 1100 nm, with Si alone, to longer than 1550 nm, covering all the telecommunication wavelengths. Furthermore, Ge exhibits higher mobility of electrons and holes, resulting in a faster detection. Therefore, Ge has long been considered as an ideal candidate to replace the conventional III-V detectors. However, the adoption of Ge is severely restricted due to the mismatch of the lattice constants between Si and Ge. The lattice mismatch poses a constraint on the maximum Ge thickness that can be grown on Si without introducing crystal defects [63]. Consequently, Ge thickness is limited for maintaining the dark current level as low as possible, resulting in a low quantum efficiency. Hence, the epitaxial growth of a thick Ge layer on Si should be the key to the realization of the SiGe PD and, more importantly, it should be achieved in a CMOS-friendly manner. 

An intuitive way to obtain a thick Ge layer with minimal dislocations is by using a graded buffer layer [64]. Based on this technique, the work in [64] achieves an extremely low dark-current level which is comparable to the theoretical reverse saturation current. Even though it completely addresses the problem of lattice mismatch, it usually results in a tall PD structure; consequently, this technique is not compatible with CMOS back-end processes and waveguide coupling. Alternatively, a direct growth of Ge on Si with the aid of a thin Ge buffer layer can be more effective [65]. Starting from a thin Ge buffer layer formed at a low temperature of 350 °C, the Ge layer can be grown to be sufficiently thick for the absorption of infrared light at a relatively high temperature of 600 °C, thus avoiding the problem of crystal defects. Thus, the detection of a wavelength of 1300 nm can be successfully achieved with a responsivity of 240 mA/W. Although not reported in this work, the dark current level is estimated to be high because the defects are not clearly eliminated. In [66], it is demonstrated that the dislocation density can be remarkably reduced by applying cyclic thermal annealing at a high temperature of 900 °C. Further extending the work in [65] by employing a cyclic thermal annealing process, the work in [67] achieves a high responsivity with a low dark-current level and the frequency response is suitable for gigabit operation. However, high-temperature annealing may not be compatible with the standard CMOS process, necessitating a different defect-handling technique for the seamless integration of the EPIC. The work in [68] demonstrates that the selective growth of Ge through multiple hydrogen annealing is also a good approach to reduce the dislocation density. 

### 3.4. Integration of PD with Waveguide

**Figure 20:**
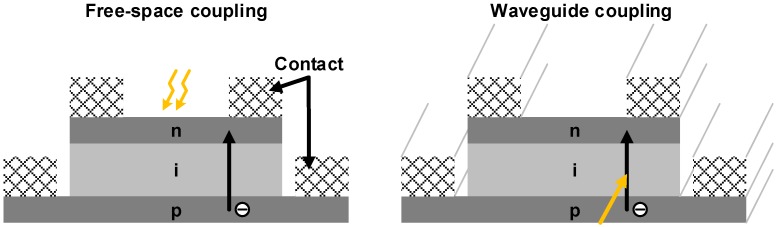
Categorization of PD by coupling schemes.

PDs can be categorized into two types according to coupling schemes: a free-space coupled PD and a waveguide-coupled PD as illustrated in Figure 20. In the case of the free-space coupled PD, a fiber can be directly coupled for a top illumination or a back illumination. In either case, the direction of the incoming light is always parallel to that of the carrier collection. Therefore, in order to enhance the quantum efficiency, the thickness of the absorption layer should be sufficiently large, which results in an increased transit time, and hence degrades the bandwidth. On the other hand, in the waveguide-coupled PD, the tradeoff between the quantum efficiency and the bandwidth can be relaxed. This is because the absorption can be improved by increasing the absorption length along the direction of the light, which does not affect the transit time. However, in this case, the increased junction capacitance may degrade the bandwidth, but not as much as in the case of the free-space coupled PD. Another benefit of the waveguide coupling is that the waveguide PD is more suitable for an on-chip WDM system, thus obviating the use of additional fibers. Evanescent coupling and butt coupling are most commonly used for waveguide coupling. Evanescent coupling is easy to realize whereas it is relatively difficult to design a structure for butt coupling. However, in terms of quantum efficiency, butt coupling with a well-designed waveguide is generally much better than evanescent coupling. 

## 4. CMOS Transimpedance Amplifier (TIA)

The TIA is the first electronic circuit that directly interfaces with a photodetector (PD) for a current-to-voltage conversion. Therefore, the overall performance of the receiver is predominantly determined by that of the TIA. There are four main parameters defining the performance of the TIA: gain, bandwidth, noise, and power. Further, these performance parameters are strongly interdependent and strong tradeoff relations exist among them. Thus, for example, we cannot improve the gain and the bandwidth simultaneously without sacrificing the power.

Traditionally, compound semiconductor devices were preferred for implementing optical interface circuits due to their high-speed capability. However, significant improvements in CMOS technologies have reduced the performance gap between these two technologies. Moreover, recently, further advancements in circuit technologies have led to CMOS implementations of extremely high-speed optical receivers whose operating speeds are higher than 40 Gb/s [53,69].

Besides enjoying the advantage of CMOS scalability, the success of CMOS-compatible silicon photonics also mandates the CMOS implementation of electronic circuits. Although most optical receivers still rely on hybrid integration, which interconnects multiple ICs from different technologies through bonding, monolithic integration is gaining increasing intention and some remarkable results have been demonstrated as previously mentioned. If realized, monolithic integration would be the best solution since it provides many benefits, such as enhanced signal integrity, small area, and reduced packaging cost, when compared to hybrid integration. In this section, we focus on CMOS realizations of the TIA by providing a brief overview of various TIA topologies reported so far.

### 4.1. Resistor-Based TIA

**Figure 21:**
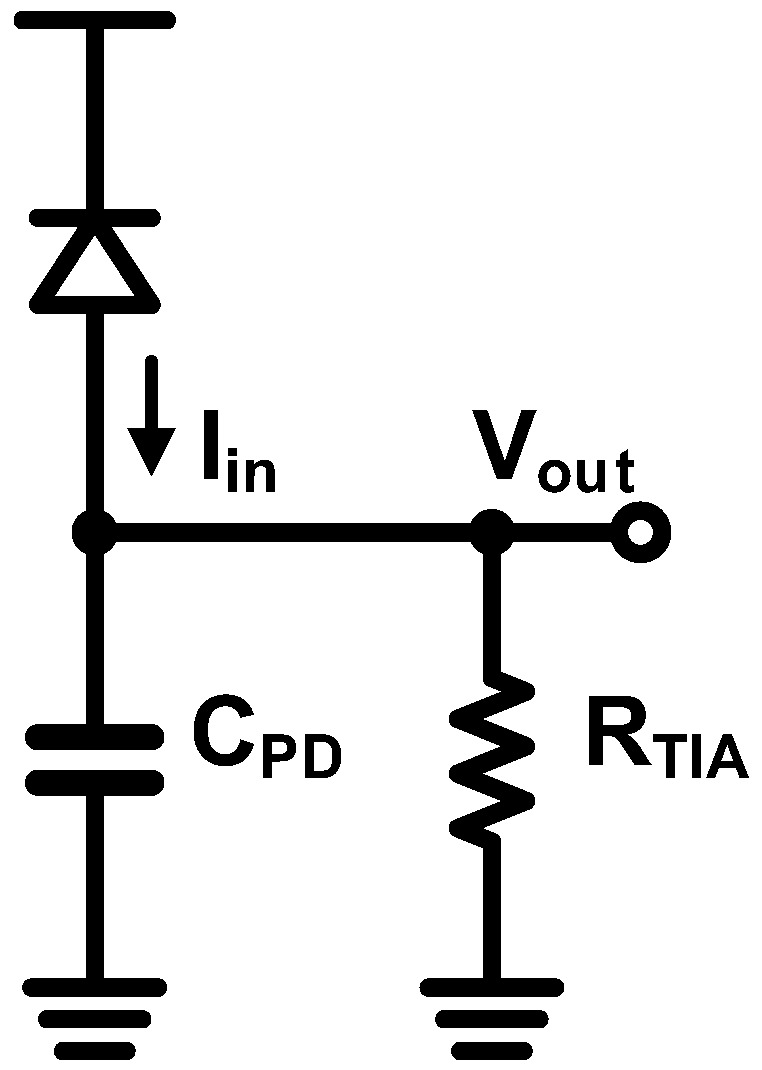
Simple implementation of TIA using single resistor.

The most intuitive way to implement the TIA is employing a single resistor as shown in Figure 21. In this configuration, the calculations of the gain, *R_T_*, and the bandwidth, *f*_−3*dB*_, are straightforward and can be expressed as follows:

(4) $$R_{T}=R_{TIA}$$

(5) $$f_{-3dB}=\frac{1}{2\pi R_{TIA}C_{PD}}$$

As evident from the above equations, there is a severe tradeoff between the gain and the bandwidth. On the other hand, the total integrated noise at the output is given by:

(6) $$\bar{V_{n,out}^{2}}=\frac{kT}{C_{PD}}$$

where *k* is the Boltzmann constant and *T* is the absolute temperature. Further, the signal-to-noise ratio (SNR) can be defined to assess the performance of the TIA as follows:

(7) $$SNR=\frac{C_{PD}}{kT}I_{in}^{2}R_{TIA}^{2}$$

which indicates that increasing *R_TIA_* enhances the SNR indefinitely. However, a large *R_TIA_* introduces an inter-symbolic interference (ISI) and thus reduces the bandwidth, which limits the maximum value of *R_TIA_* for a given *C_PD_*. In general, the minimum bandwidth sufficiently suppressing the ISI is chosen to be 0.5–0.7 times the target data rate. Thus, the only way to enhance the SNR is increasing the input optical power, thereby providing a large input current. Furthermore, a large *C_PD_* renders this type of TIAs unsuitable for high-speed optical receivers. Nevertheless, this type of TIAs exhibits a distinct advantage in that it consumes virtually zero power. Recently, by effectively cancelling the ISI, a resistor-based TIA is successfully demonstrated at moderately high speed with low power consumption [70].

### 4.2. Common-Gate-Based TIA

As discussed in the previous section, a resistor-based TIA suffers badly from the gain-bandwidth tradeoff, which results in a limited achievable SNR. In order to overcome the disadvantage, a common-gate (CG) topology has been widely used. 

**Figure 22:**
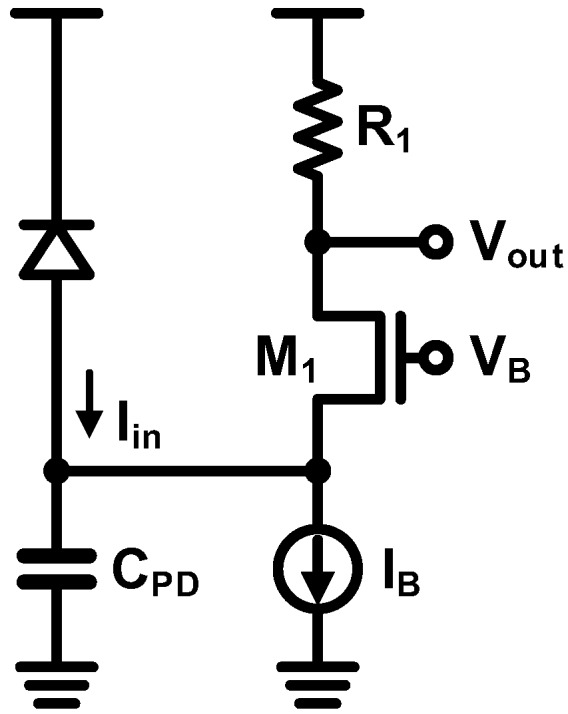
Common-gate TIA.

The basic CG amplifier as a TIA is illustrated in Figure 22. Assuming a dominant pole is located at the input, the gain, *R_T_*, and the bandwidth, *f*_−3*dB*_, are expressed as follows:

(8) $$R_{T}=R_{1}$$

(9) $$f_{-3dB}=\frac{g_{m1}}{2\pi C_{PD}}$$

Note that the gain-bandwidth tradeoff is now completely eliminated, indicating that the gain can be improved without degrading the bandwidth significantly. 

**Figure 23:**
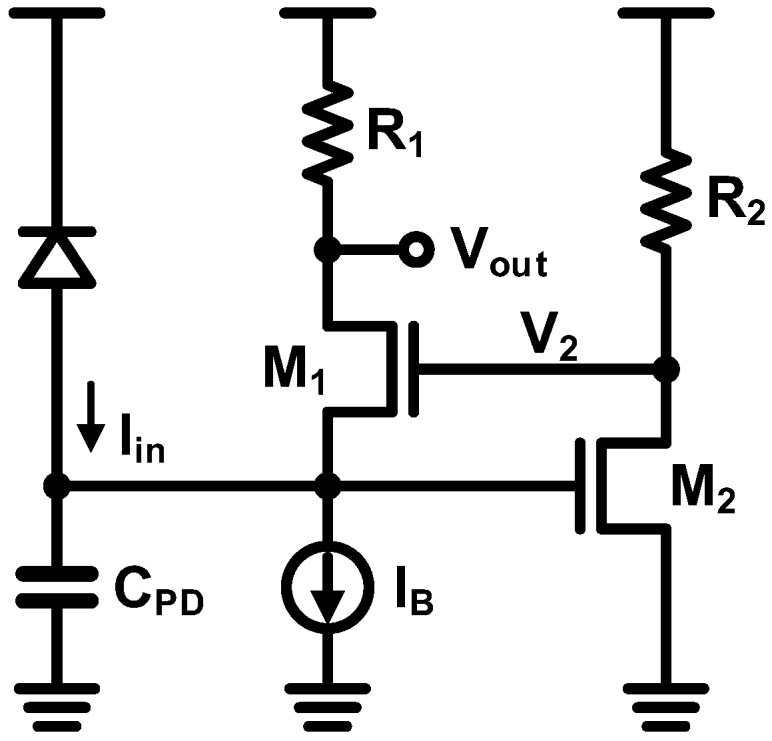
Regulated-cascode TIA.

Even if the SNR can be enhanced by employing the CG TIA, a direct tradeoff between the bandwidth and the power consumption exists, which limits the use of the CG TIA in the present configuration. Alternatively, a regulated-cascode (RGC) TIA shown in Figure 23 can further improve the bandwidth by lowering the input resistance [71]. The gain of the RGC TIA is the same as that of the CG TIA and the bandwidth is given by (with the same dominant-pole condition):

(10) $$f_{-3dB}=\frac{g_{m1}\left(1+g_{m2}R_{2}\right)}{2\pi C_{PD}}$$

With linearly increasing *I_B_*, *g_m_* and thus *f*_−3*dB*_ are increased approximately by the square of *g_m_*, thereby alleviating the direct tradeoff between bandwidth and power consumption. Owing to their clear advantage, many TIAs based on the RGC topology have been proposed. The bandwidth of the RGC TIA can be further enhanced by combining a shunt-shunt feedback [72] and a differential RGC TIA can also be configured [73]. Recently, RGC-based TIAs have been successfully demonstrated with operating speed higher than 25 Gb/s [54,56,74].

Despite the advantages of the RGC TIA, it is not suitable in the recent CMOS trend wherein the supply voltage is aggressively scaled down, because it suffers from a small voltage headroom due to stacking of the two NMOS transistors. Specifically, the output voltage, *V_out_*, in Figure 23 has to accommodate the gate-source voltage of M_2_, the drain-source voltage of M_1_, and the voltage across R_1_. The output of the common-source (CS) amplifier, *V_2_*, also has to accommodate the gate-source voltages of M_1_ and M_2_, and the voltage across R_2_. These conditions render the RGC TIA inapplicable to sub-1 V CMOS technologies. 

**Figure 24:**
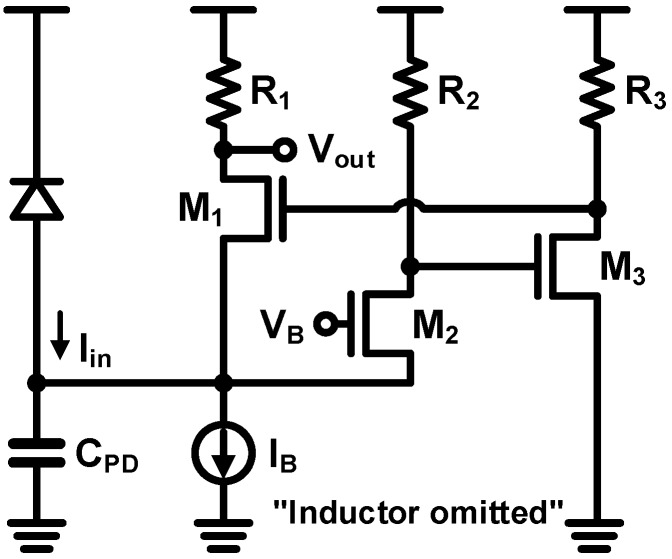
CG-feedforward TIA.

As an alternative, a CG-feedforward TIA illustrated in Figure 24 is proposed in [75], achieving a wider bandwidth and relaxing the voltage headroom simultaneously. Furthermore, the gain of the CG feedforward TIA is approximately the same as that of the CG and the RGC TIA. The bandwidth can be calculated as:

(11) $$f_{-3dB}=\frac{g_{m1}\left(1+g_{m2}g_{m3}R_{2}R_{3}\right)}{2\pi C_{PD}}$$

The bandwidth is significantly enhanced than the RGC TIA and the voltage-headroom problems are also greatly mitigated. By employing this topology, the work in [75] achieves a considerably high bandwidth of 20 GHz in the standard CMOS platform.

### 4.3. Feedback-Based TIA

**Figure 25:**
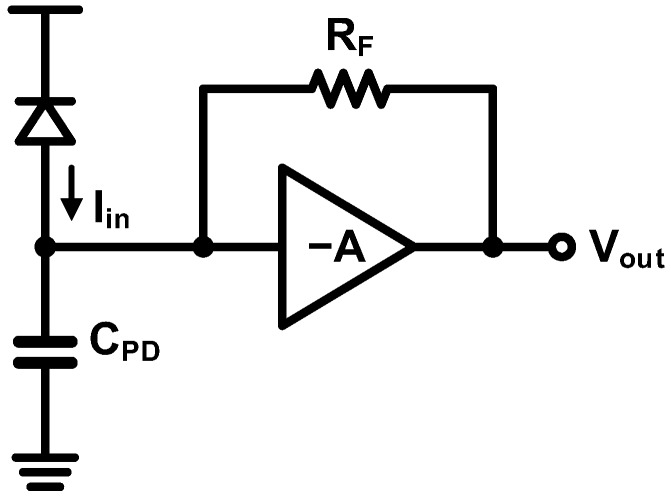
Shunt-shunt feedback TIA.

Along with the CG-based TIAs, a shunt-shunt feedback TIA has been widely employed in optical receivers. Figure 25 shows a basic feedback-based TIA topology. With an ideal amplifier, the gain and the bandwidth can be approximated as follows:

(12) $$R_{T}=R_{F}$$

(13) $$f_{-3dB}=\frac{1+A}{2\pi R_{F}C_{PD}}$$

As (13) indicates, the bandwidth can be enhanced by the higher gain of the amplifier. Therefore, designing an amplifier that exhibits a high gain and a low output impedance is crucial for achieving good performance. In general, this type of TIAs is more advantageous than the CG-based topology in terms of voltage headroom and features a relatively simple architecture favorable for optimization.

**Figure 26:**
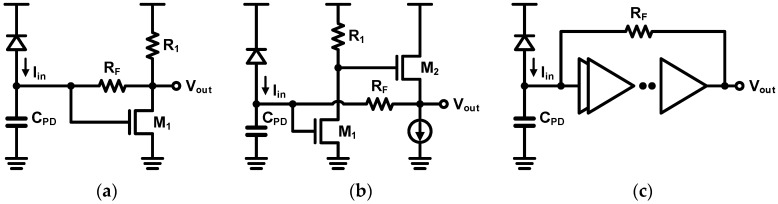
Various implementations of shunt-shunt feedback TIA. (**a**) CS amplifier with resistor feedback, (**b**) Cascaded CS amplifier and source follower with resistor feedback, and (**c**) multi-stage amplifier with resistor feedback.

As shown in Figure 26, a variety of versions of the feedback-based TIA have been attempted with different implementations of the amplifier. Figure 26a illustrates a traditional TIA implementation which was first demonstrated with CMOS technology in [76] and achieved 1-Gb/s operation. This topology has been widely used and has succeeded in achieving high-speed operations of 10 and 25 Gb/s [44,77]. Alternatively, as shown in Figure 26b, the combination of a common-source amplifier, a source follower, and a feedback resistor has been commonly employed to provide a low output impedance [78,79,80]. Especially, the work in [80] achieves a 10-Gb/s operation by combining this topology with bandwidth-enhancement techniques. In order to achieve a higher gain, the feedback can be applied to a multi-stage amplifier as illustrated in Figure 26c. In [81], the feedback resistor is connected between the input and the output of the three-stage inverter amplifier. The feedback can also be applied to the differential topology as implemented in [82,83] with two-stage and four-stage amplifiers, respectively. In spite of several advantages of the feedback-based TIAs, they suffer from an inherent stability issue which is particularly evident in the multi-stage topologies.

### 4.4. Inverter-Based TIA

**Figure 27:**
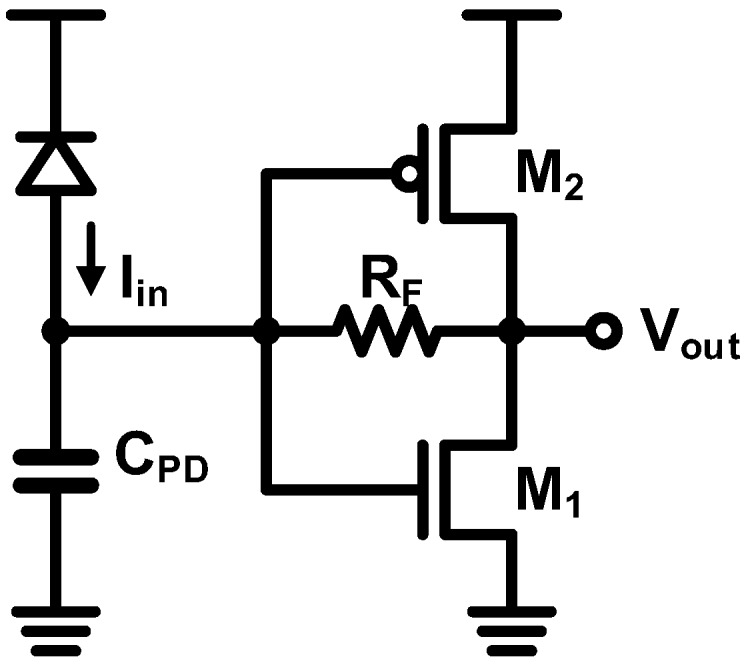
Inverter-based TIA.

Based on the shunt-shunt feedback, an inverter-based TIA is currently the most popular topology and it was first implemented with a CMOS technology for a 1-Gb/s operation 20 years ago [84]. As shown in Figure 27, the inverter-based TIA features a very simple architecture consisting only of a CMOS inverter and a feedback resistor. Even if the bandwidth enhancement factor is limited to the gain of the one-stage inverter, this topology has numerous advantages over its competitors. First, owing to its simple architecture, the voltage headroom is significantly mitigated and no biasing circuit is needed. Second, since it can exploit *g_m_* of both the NMOS and the PMOS, high *g_m_* can be achieved with considerably low power consumption [52]. Nevertheless, the inverter-based TIA has not been frequently used, because it is not compatible with the traditional technologies. However, there is a major contributor to the widespread use of this topology. The CMOS technology continues to be developed in such a way that most of the performance are optimized for digital circuits, but not for analog counterparts, thereby forcing the inverter-based TIA to be the most suitable topology for modern CMOS technologies. Therefore, after being revisited in [52], the inverter-based TIA is now widely employed for high-speed and low-power applications [7,43,50,55,69,85,86,87,88,89,90,91]. In the state-of-the-art implementation, the optical receiver based on this topology demonstrates a 64-Gb/s operation, which is recorded as the fastest speed ever achieved in the CMOS platform [53].

### 4.5. Integrating Receiver

**Figure 28:**
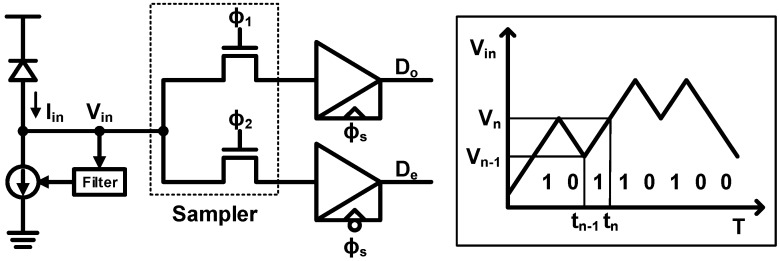
Integrating receiver based on double sampling.

The aforementioned topologies are based on an amplifier which offers a high-bandwidth and low-noise characteristics generally at the cost of an increased power consumption. In order to overcome this, an integrating receiver is proposed using a different method for achieving a high sensitivity with a low-power consumption [92]. The basic concept is briefly explained in Figure 28. The photocurrent is first integrated by a sampler using two non-overlapping phases, thus charging or discharging *V_in_* according to the incoming data. After the integrating phase, the current value, *V_n_*, is compared with the previous value, *V_n_*_−1_, to make a proper decision. Based on this scheme, the work in [92] achieves a 1.6-Gb/s operation while consuming considerably low power. The works in [93,94] further extend this work, and achieve much higher operation speeds of 16 and 24 Gb/s, respectively.

## 5. Bandwidth Extension Techniques

In the CMOS front-end for photodetection, there are many parasitic capacitances that limit the circuit bandwidth, such as the input capacitance of PD, parasitic capacitances of CMOS devices, and output loading of TIAs. Therefore, as the required communication bandwidth for optical links increases, CMOS circuits have become a bottleneck for achieving such high bandwidth. Several circuit techniques for bandwidth extension of TIAs and optical receivers have been presented in the literature [80,85,95,96,97,98,99,100]. The techniques are classified into two categories: an inductive peaking technique and an equalization technique. In this section, a brief summary and categorization of various bandwidth extension techniques are presented.

### 5.1. Inductive Peaking

**Figure 29:**
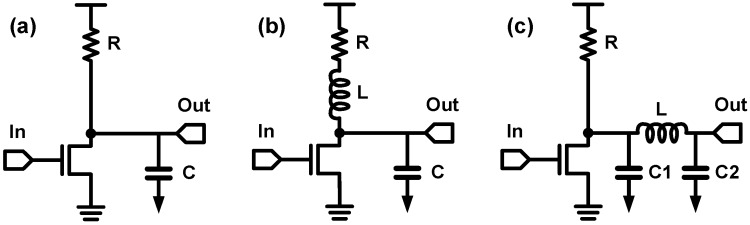
Circuit diagrams of (**a**) CS stage without inductive peaking, (**b**) CS stage with shunt peaking, (**c**) and CS stage with series peaking.

In this subsection, the basic principles of inductive peaking techniques are briefed and examples of CMOS photodetection circuits that adopt the peaking techniques are described. Inductive peaking technique has a long history and its integration into CMOS technology is well evaluated in the literature [101,102,103]. Nevertheless, an integrated inductor occupies a huge silicon area and thus increases the cost of the IC significantly. Therefore, the use of inductors in a photo-detecting IC should be considered carefully and the inductors should be optimized precisely. Fortunately, there are a lot of novel inductive peaking techniques, which have been developed to overcome the bandwidth limit of the legacy electrical link or ultra-wideband amplifier. Some of them have been adopted in high-speed photodetection circuits during the last two decades. Basically, there are two types of inductive peaking—shunt peaking and series peaking—and their examples with a simple CS stage are shown in Figure 29. Without inductive peaking, the transfer function of the CS stage is:

(14) $$H\left(s\right)=\frac{g_{m}R}{1+\mathrm{sRC}}$$

which is a simple one-pole system. On the other hand, for the shunt-inductive peaking, which is shown in Figure 29b, the inductor introduces a zero and an additional pole in the transfer function so that the transfer function becomes:

(15) $$H\left(s\right)=\frac{g_{m}\left(R+\mathrm{sL}\right)}{1+\mathrm{sRC}+s^{2}\mathrm{LC}}$$

By placing the zero properly in accordance with the positions of the poles, the zero can compensate the dominant pole, and therefore, it improves the circuit bandwidth [102]. From a qualitative point of view, the inductor blocks the high-frequency current flowing through the resistor so that most of the bias current is solely used for charging/discharging the load capacitor, whereas the bias current is divided into the resistor and the load capacitor in the normal CS stage. Therefore, the rise/fall time reduces, which indicates that the circuit bandwidth is enhanced. The quantitative details of the bandwidth enhancement of the shunt peaking are presented in [102], and it is demonstrated that the maximum bandwidth enhancement ratio (BWER) of the shunt peaking is 1.84 with 1.5 dB peaking in magnitude response. The BWER is defined as the ratio of the 3-dB bandwidth with an inductive peaking to that without peaking. On the other hand, it is slightly more difficult to understand the principle of the series-inductive peaking in the s-domain model as compared to the shunt peaking because the inductor and the separation of the load capacitor introduce two additional poles but do not introduce any zero. Since there are three poles without a zero, the bandwidth extension is dominated by the damping factor of the transfer function, which is difficult to observe intuitively. On the other hand, a qualitative approach provides a much simple explanation. Since the high-frequency current is blocked by the series inductor, *C1* and *C2* are charged sequentially. Extremely, *C2* is charged after *C1* is fully charged. In other words, the rise/fall time of the signal is reduced as a function of the ratio of *C1* to *C2*, at the expense of increased delay [103]. The BWER of the series peaking can be higher than 2.5 with a specific ratio of *C1* over *C2* [102]. 

**Figure 30:**
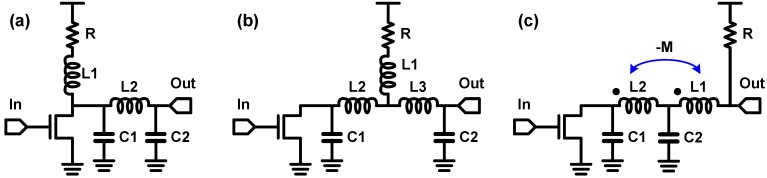
Circuit diagrams of (**a**) shunt and series peaking, (**b**) shunt and double-series peaking, (**c**) and T-coil peaking network.

In Figure 30, some advanced inductive peaking techniques are illustrated. Figure 30a shows a shunt-and-series peaking, which combines the shunt peaking and the series peaking in Figure 29. In 2004, a UCLA group proposed to employ this technique for a post-amplifier preceded by an optical TIA and achieved a BWER of 3.5 in [104]. For reference, they used different terminology in their paper; they named this technique the triple-resonance architecture. The inductive peaking technique shown in Figure 30b is widely referred to as the shunt and double series peaking. By placing an additional inductor (*L3*) over the shunt and series peaking, better isolation of the capacitors can be obtained; hence, the circuit bandwidth can be further enhanced [103]. However, this technique requires three inductors, thus occupying extensive silicon area. A T-coil network shown in Figure 30c resolves this challenge [97,101,103]. The negative coupling between the two inductors (*L1* and *L2*) affects the initial boost in the current flow to *C2*, which indicates that *C2* is effectively connected in series with the negative mutual inductance element of the T-coil [102]. In other words, the T-coil network is equivalent to the shunt and double series peaking in Figure 30b, where three inductors form a ‘T’ shape. That is why this technique is called a ‘T-coil’ network. With the T-coil network, the number of inductors is reduced to two. Moreover, the two inductors or the transformer can be implemented in silicon IC while occupying lesser area than the two inductors without magnetic coupling, by using novel winding techniques for T-coils [102,103]. The achievable BWER of T-coil network is approximately 4.0 with a reasonable *C1* over *C2* ratio and gain peaking, and the detailed quantitative values of BWER of the T-coil network are provided in [102]. 

**Figure 31:**
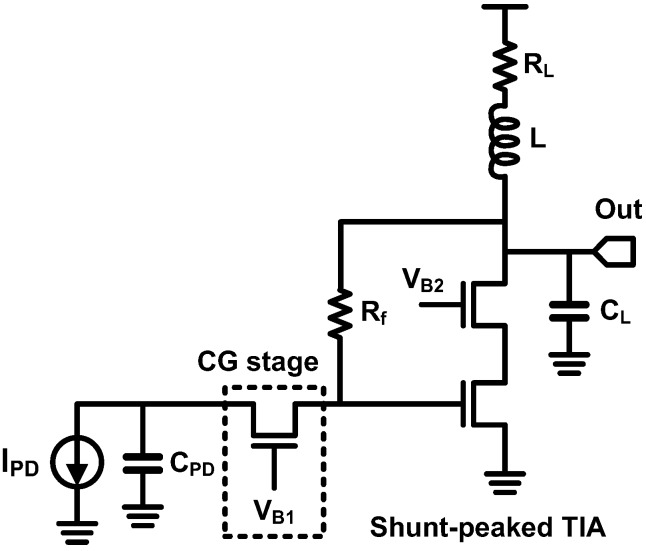
TIA with shunt peaking presented in [80].

From now on, the inductive peaking techniques employed in the TIAs are introduced. In 2000, a Stanford group introduced the shunt inductive peaking into their TIA based on a resistive feedback CS stage as shown in Figure 31 [95]. A CG stage is placed between the shunt-peaked CS stage and the PD for decoupling the PD capacitance and the main stage and for introducing an additional flexibility to optimize the shunt inductor. With the proposed technique, they achieved BWER of 1.40 using an on-chip inductor of 20 nH. Moreover, it was verified that their TIA achieves a bandwidth of 1.2 GHz when fabricated in 0.5-µm CMOS technology. To the best of the authors’ knowledge, the first introduction of the series inductive peaking into high-speed photodetections was performed by a Caltech research group in 2004 [80]. Their TIA, which was fabricated in 0.18-µm CMOS, consists of three gain stages in order to achieve sufficient transimpedance gain and includes four series inductors.

**Figure 32:**
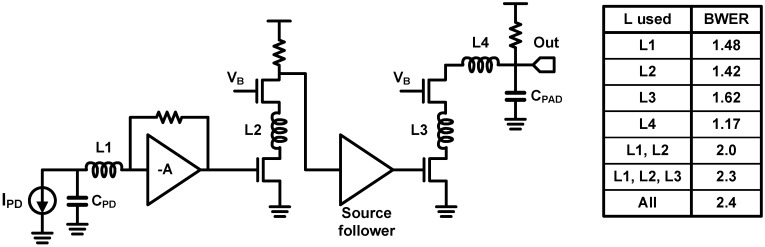
Three-stage TIA with series peaking presented in [65].

**Figure 33:**
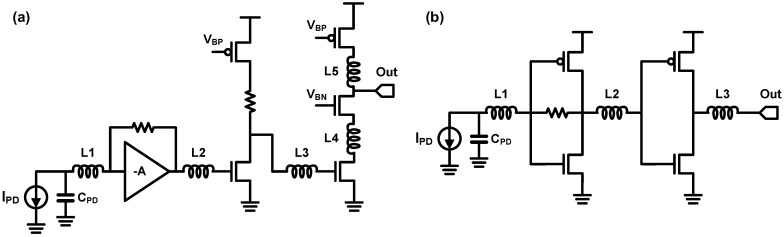
TIA examples with series peaking between cascaded gain stages presented in (**a**) [61] and (**b**) [71].

**Figure 34:**
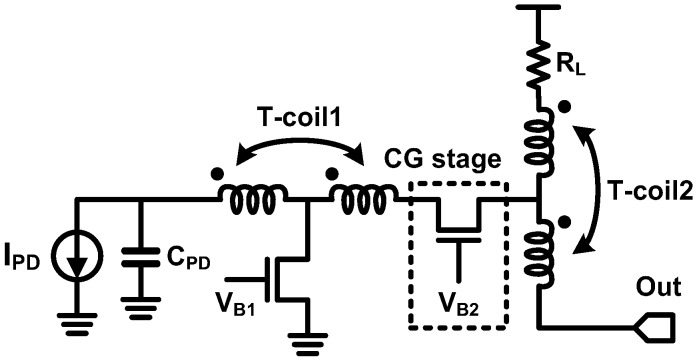
CG TIA with T-coil peaking presented in [82].

As shown in Figure 32, each series inductor separates the adjacent capacitors as follows: (1) *L1*: the PD capacitance and the TIA input capacitance, (2) *L2* and *L3*: the junction capacitances of cascode transistors, and (3) *L4*: the output parasitic capacitance/bonding pad capacitance. The four series inductors achieve an overall BWER of 2.4, and each contribution of the inductors is summarized in Figure 32. The 3-dB bandwidth of the TIA is 9.2 GHz and the eye-diagram at 10 Gb/s is verified. In addition to using series inductors between cascode transistors, a UCSD group also added series inductors between the cascaded gain stages [98]. Two series inductors, *L2* and *L3*, are placed between the cascaded amplifiers for further bandwidth extension while adopting a similar structure with [80], as shown in Figure 33. Moreover, it is notable that the authors of [98] did not overlook the degradation of phase linearity owing to the increased BWER. They evaluated the group delay response of the TIA as well as the BWER; thus, a group delay variation of 16 ps was achieved for their TIA whereas that of the TIA in [80] exceeds 50 ps. As a result, while fabricated in 0.13-µm CMOS, the TIA opens the eye diagram at 40 Gb/s and achieves better eye opening compared to [80]. In 2012, the same group proposed to combine the series peaking technique with the inverter-based TIA, which is more appropriate for use in deep sub-micron CMOS SOI technology and with a lower supply voltage [69]. With a 1.0-V supply voltage, the TIA achieves 3-dB bandwidth of 30 GHz while dissipating 9 mW. They also emphasized the important of optimizing the phase response, and they attempted to suppress the group delay variation for better phase response. Therefore, the group delay variation was suppressed to less than 8 ps, thus achieving better eye opening compared to their previous work in [98]. T-coil peaking technique was introduced in TIA design by Ehwa Womans University in 2010 [97]. As shown in Figure 34, CG topology was chosen and two T-coil networks were employed to extend the bandwidth. The first T-coil network (T-coil1) separates *C_PD_* from the junction capacitances of the transistors, and the second T-coil network (T-coil2) provides shunt-peaking with *R_L_* and separates the junction capacitance and the output load capacitance. BWER of 2.3 and 3-dB bandwidth of 12.6 GHz were achieved, whereas the phase response was not fully optimized. 

**Figure 35:**
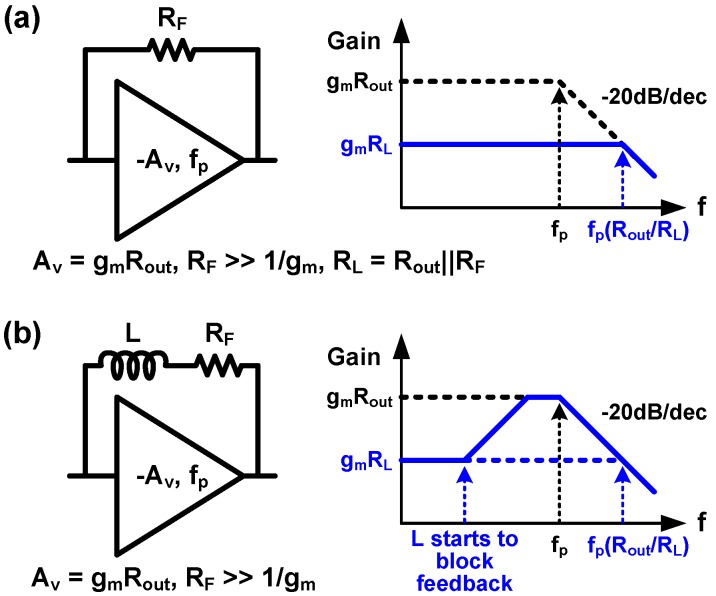
Effect of feedback on the transfer function of an amplifier (**a**) with only resistive feedback (**b**) with the combination of resistive and inductive feedback.

In addition to the inductive peaking techniques shown in Figure 29 and Figure 30, an inductive feedback technique wherein an inductor is placed in series with a feedback resistor of a TIA has been proposed in the literature [44,100,105]. A straightforward intuition of the bandwidth extension using inductive feedback is summarized in Figure 35, where *R_F_* and *L* denote the feedback resistance and inductance, respectively. Assuming a simple CS amplifier whose gain and 3-dB bandwidth are -*g_m_R_out_* and *f_p_* respectively, a resistive feedback improves the bandwidth by a factor of *R_out_/R_L_*, where *R_L_* is the resistance of the parallel combination of *R_out_* and *R_F_*, whereas the gain is reduced by the same factor, as shown in Figure 35a. On the other hand, insulting a series inductor to *R_F_*, as shown in Figure 35b, results in peaking as well as the bandwidth extension. At a low frequency, since the impedance of the inductor is almost zero so that it can be neglected, the transfer function with the inductor becomes the same as that without inductor. 

However, the impedance of the inductor increases as the frequency increases; hence, it becomes larger than that of *R_F_* at a certain frequency, which can be considered as a zero frequency. This indicates that the feedback strength decreases, and consequently, the transfer function begins to follow that without the resistive feedback. In other words, when the inductance dominates the overall feedback impedance, the feedback network becomes negligible; i.e., the transfer function exhibits a peaking as shown in Figure 35b, which follows the low-gain curve at a low frequency and the high-gain curve at a high frequency.

Luxtera Inc. adopted the inductive feedback technique in the CS-stage-based TIA in 2006 [44]. They also reported that the inductor reduces the input-referred noise caused by the feedback resistor. In [100,105], the inductive feedback was employed in the inverter-based TIA by the authors’ group, in order to compensate the frequency dependent loss owing to the PD bonding parasitic impedance by utilizing the peaking illustrated in Figure 35. With inductive peaking, the 3-dB bandwidth of the TIA extends from 11.4 GHz to 25.2 GHz, which corresponds to a BWER of 2.2 dB.

There is an interesting inductive peaking technique proposed by TSMC Inc. in 2014, which is called a shared inductor [85]. When an inductor is shared between adjacent amplifier stages, the number of inductors used in the photodetecting circuit halves; moreover, the size of each inductor can be further reduced. The fabrication in 28-nm CMOS technology is provided in [85], and it was reported that the overall chip area reduced by 56% in addition to a power saving of 27% as compared to the fabricated prototype chip with the conventional inductive peaking. Since the on-chip inductor occupies a large area in ICs, the shared inductor is a notable circuit technique because it can significantly reduce the chip area occupied by inductors.

### 5.2. Equalization

In the electrical link receiver, various types of equalization techniques have been utilized to overcome the frequency dependent loss of the electrical transmission line. On the other hand, there has been little interest in using such equalization techniques for optical links because optical fibers provide almost infinite transmission bandwidth. However, equalization techniques have been adopted in recent state-of-the-art optical receivers in order to (1) overcome the limited O/E converting speed of PD, (2) break the tradeoff between high-gain and high-bandwidth, and (3) enhance the TIA bandwidth with minimal added noise. This section summarizes the equalization techniques for a photodetecting circuit that have been presented in leading journals and conferences recently.

**Figure 36:**
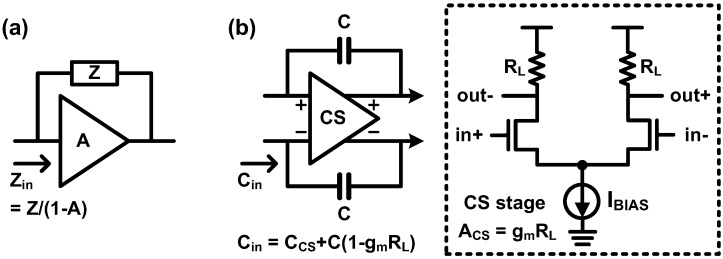
(**a**) Miller effect (**b**) Miller capacitance applied in a differential CS stage in [91].

In 2010, a University of Toronto group proposed a TIA with negative Miller capacitances and a continuous-time linear equalizer (CTLE) [106] in order to overcome the limited speed of a spatially modulated PD. Figure 36 shows the principle of the Miller effect. In definition, the impedance is the coefficient of the voltage difference between the two ports of a device under test (DUT) over the current flowing through the DUT. In case there is an amplifier whose input and output are connected to the two ports of a DUT, the voltage applied to the DUT is influenced by the amplifier as well as the input voltage. Therefore, the amplifier gain should be included in the impedance. For example, the input impedance becomes *Z*/(1-*A*), where *Z* and *A* represent the original impedance of the DUT and gain of the amplifier, respectively. It can be achieved by using the Kirchhoff’s current law (KCL) at the input node, as long as the amplifier sets the ratio of the output voltage over the input voltage to *A*. Because the Miller effect is a truly well-known circuit effect, further explanations can be found in many textbooks, i.e. [107]. In [106], a pair of capacitors is placed between the input and output of a differential CS stage TIA, which have the same polarity, as shown in Figure 36b. Owing to the Miller effect, the input capacitance of the TIA becomes *C_CS_* + *C*(1-*A_CS_*), where *C_CS_*, *C*, and *A_CS_* are the input capacitance of the CS stage, Miller capacitance, and gain of the CS stage, respectively. Notably, the second capacitance term of the Miller capacitance has a negative value when the gain of the CS stage is larger than unity. That is why this technique is referred to as the negative Miller capacitance. Accordingly, the input capacitance becomes lower than *C_CS_* as long as the gain of the CS stage is larger than unity, thus improving the bandwidth of the preceding amplifier.

**Figure 37:**
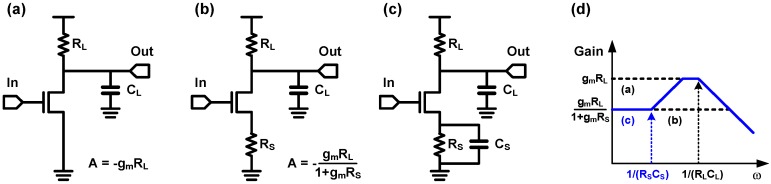
Summary of CTLE based on source degenerated CS stage. (**a**–**c**) Circuit diagrams of normal CS stage, CS stage with source degeneration resistor, and CS stage with source degeneration resistor and capacitor (CTLE). (**d**) Transfer function of CTLE.

**Figure 38:**
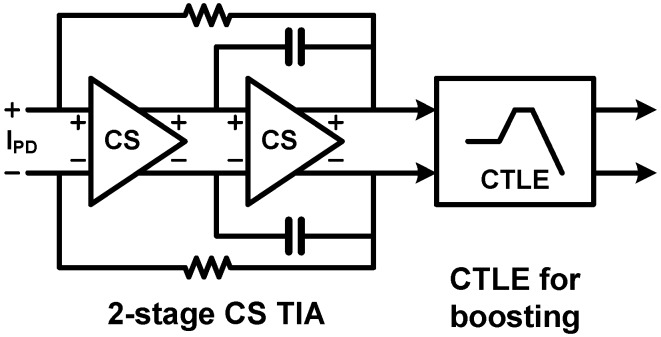
Overall structure of [91].

A CTLE introduces a similar effect with the inductive feedback technique discussed in the previous section. A CTLE is generally implemented with a CS stage with source degeneration using a combination of a resistor and a capacitor. As shown in Figure 37, the source degeneration with a resistor (*R_S_* in Figure 37b) decreases the gain of the CS stage by a factor of (1 + *g_m_R_S_*) while the circuit bandwidth is extended by the same factor [107]. By placing a degeneration capacitor (*C_S_*) in parallel with *R_S_*, the degeneration strength becomes weakened at a high frequency so that the transfer function follows that of the normal CS stage, which is similar to the effect of the inductive feedback. As a result, the CTLE achieves a high-frequency peaking in its transfer function as shown in Figure 37d. The overall photodetecting circuit presented in [106] is shown in Figure 38. Two identical CS stages are cascaded for high gain, and the negative Miller capacitance is used for the second stage to reduce the load capacitance driven by the first CS stage. After the cascaded CS stage, CTLE is used to compensate the high-frequency loss owing to the limited bandwidth of a spatially modulated PD, which is monolithically integrated in a single chip. Therefore, the work in [106] achieves 5-Gb/s data rate while the 3-dB bandwidth of the spatially modulated PD is only 700 MHz.

**Figure 39:**
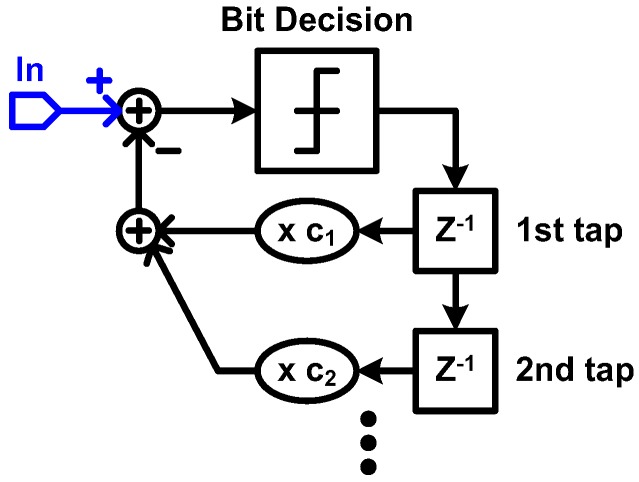
Simplified block diagram of DFE.

**Figure 40:**
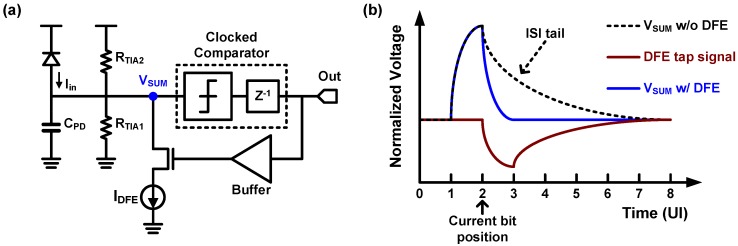
Resistor-based TIA with IIR-DFE proposed in [55].

In order to achieve sufficient high-frequency boosting and to overcome noise amplification of CTLE, a non-linear equalization technique of a decision feedback equalizer (DFE) has been introduced for optical receivers. The DFE technique has been widely used in electrical links to compensate for inter-symbol interference (ISI) caused by the frequency dependent loss and reflections [9,25,108,109,110]. Because a theoretical background of DFE can be found in many publications [9,111,112,113,114], only a brief introduction is provided in this paper for better clarification of the focus. Owing to the loss and reflections, a single transmitted bit cannot complete its transition within a bit period or a unit-interval (UI) so that it influences the following bit sequence. The basic concept of the DFE is that the previously received bit sequence is used when a receiver makes a decision whether the currently received bit is ‘zero’ or ‘one’, by feeding back the previously decided bit sequence to the input as shown in Figure 39. That is, the DFE uses consecutive multiple bits to distinguish a single bit because the bits interact with each other due to the ISI. The degree of compensation is closely related to the number of DFE taps, which determines the length of the previous bit sequence to be included in the decision of the current bit. However, the power consumption of DFE circuitry is proportional to the number of taps. This indicates that the DFE requires large power consumption to achieve a sufficient compensation, which renders it less attractive to adopt the DFE for optical links. In 2013, however, an IBM Research group proposed an optical receiver by employing the DFE with infinite impulse response (IIR) feedback [70]. Its most interesting feature is that the receiver uses a resistor-based TIA for *I–V* conversion as shown in Figure 40a. As mentioned earlier, the resistor-based TIA should sacrifice the circuit bandwidth for gain and SNR. In this approach, a large resistor is used to achieve high gain and SNR at the expense of reduced bandwidth, and the resulting ISI is compensated by DFE. Moreover, only one tap DFE is used to compensate the ISI, which arises from a nearly infinite number of preceding bits, by using IIR feedback, and therefore, the overhead of a large number of DFE taps is significantly reduced. In contrast to electrical links where the ISI is mainly caused by external components so that is difficult to predict, the RC time constant between the PD capacitance and the resistor, which is located in the IC and is truly predictable, determines the ISI in a resistor-based receiver. As a result, by making the first feedback tap experience the same RC time constant as the input signal, the feedback signal is exponentially decayed in the same manner as the currently incoming signal so that it can compensate infinite taps of ISI as shown in Figure 40b. With the IIR-DFE technique, the resistor-based TIA achieves a data rate of 9 Gb/s. In [115], a University of Toronto group extended the adoption of IIR-DFE to the RGC TIA structure, where the dominant pole is located at the output of the TIA. 

Compared with [70], the dominant pole is moved from the TIA input to the TIA output; therefore, the DFE feedback subtraction is placed at the output of the TIA in [115]. A data rate of 20 Gb/s is achieved without using any inductive peaking technique with a relative small area of 0.027 mm^2^.

## 6. Clock and Data Recovery (CDR) Circuits

### 6.1. CDR Basic

**Figure 41:**
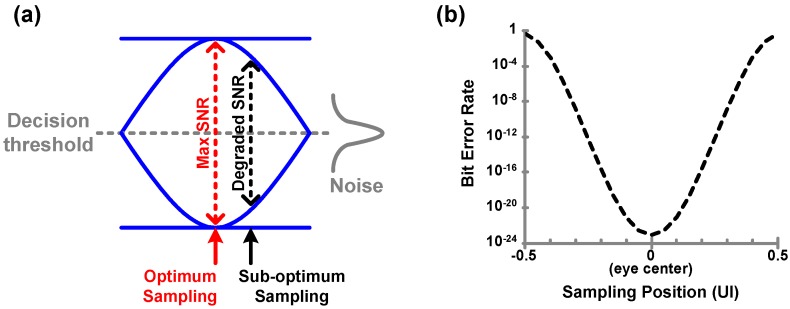
Dependency of BER on sampling timing. (**a**) Qualitative explanation of BER degradation and (**b**) quantitative relation of BER with sampling timing using the formula in [105].

Because the transmission of binary data in optical communications is based on a time-division multiplexing [116], just amplifying the incoming signal is not sufficient to recover the data [93]. The other requirement for the complete recovery of the data is to retime the incoming data with a clock whose frequency is well-aligned with the bit-rate of the data. In general, the retiming operation is based on sample-and-regeneration using a clocked sense amplifier [117,118]. That is, the timing information of the data is recovered by sampling, while the value of binary data is gathered by sampling and recovered to digital rail-to-rail amplitude by regenerating. Notably, the SNR at the sense amplifier input is restored after being regenerated at the sense amplifier output, unlike linear amplifiers where the input SNR propagates to the output, because the regeneration is based on the positive feedback. Rather, the input SNR leads to a probability of wrong decisions while the output has little noise component. Therefore, during the retiming operation, it is very important to sample the data at a proper time at which the SNR is maximized. Unlike the retiming of the digital signal domain, where only the requirements of the setup and hold time are considered, the error rate from the retiming of analog domain varies with the timing, even though the timing satisfies the setup and hold time requirements. Figure 41a illustrates this aspect. In [119], the theoretical dependency of BER on the sampling timing is analyzed. The calculated BER as a function of the sampling timing using the formula provided in [119] is shown in Figure 41b, where a maximum SNR of 10 is used for the calculation. For the sake of simplicity, it is assumed that there is no random timing error, which is referred to as a jitter. As observed in Figure 41b, the BER is exponentially degraded as the sampling timing alters from the ideal position. A CDR circuit is used as the building block of an optical receiver, and it generates the sampling clock with a precise frequency and drives the sampling timing to the ideal position. In conventional optical receivers, the importance of CDR has been underestimated or even ignored for a long time [93]. However, recently, there have been some engineering examples that include the CDR circuits for better performance and robustness. This section provides the CDR circuit examples used in a high-speed photodetection receiver. 

### 6.2. CDR Examples

**Figure 42:**
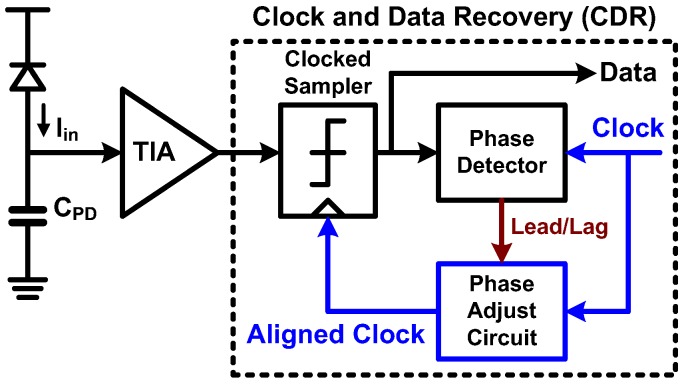
Simplified block diagram of a complete high-speed photodetecting receiver including CDR.

Figure 42 shows a block diagram of a receiver, which includes CDR circuitry. First, a TIA converts and amplifies the input current-mode signal from PD into a voltage-mode signal. Subsequently, a clocked sampler such as a StrongArm latch retimes the voltage-mode signal with the sampling clock and also regenerates the signal to digital rail-to-rail swing. The operation principles and characterization of the clocked samplers can be found in [118]. As mentioned above, the frequency of the sampling clock should be well-aligned with the bit-rate of the incoming data. Depending on the applications and architectures, the sampling clock can be generated within the CDR circuit and can also be provided from a transmitter or a global clock generation circuit. The categorization and analysis on the clocking architecture are provided in [120,121]. The relative phase of the sampled data and the clock is compared in the phase detector circuit. According to the relative phase information obtained from the phase detector, a phase adjusting circuit aligns the sampling timing to the optimum position, which is the center of the eye in Figure 41.

**Figure 43:**
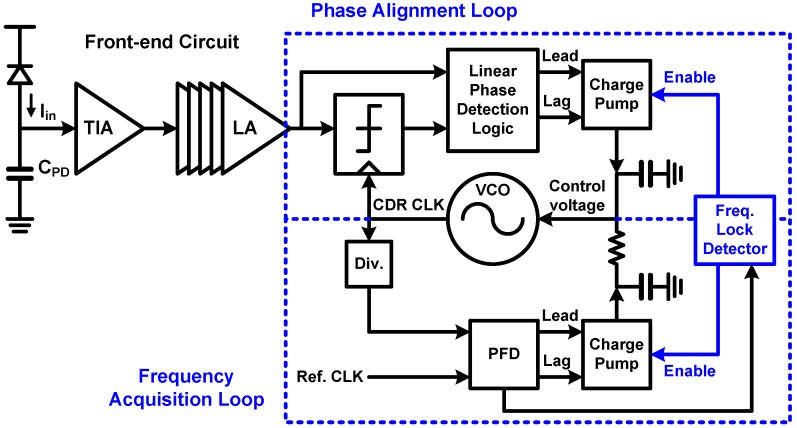
Sequential locking CDR presented in [29].

The first CDR example to be introduced was presented in [44] by Luxtera Inc. in 2006. As shown in Figure 43, it consists of two negative feedback loops: one for the precise phase alignment and the other for the initial frequency acquisition. In order to avoid interference between the loops, a locking procedure that locks the loops sequentially is used in the CDR. At the initial stage, the frequency acquisition loop is enabled until a frequency lock detector determines that the frequency of the CDR clock is sufficiently close to the data rate of the incoming data using an externally provided reference frequency. Subsequently, the CDR enters the normal CDR operation state, where the lock detector activates the phase alignment loop and simultaneously disables the frequency acquisition loop in order to track the optimum sampling phase. Although the frequency acquisition loop is disabled in the normal operation, the residual frequency error can be tracked only with the phase alignment loop, as long as the frequency error is within the lock-in range [122,123]. 

If a large frequency error out of the lock-in range is introduced, the lock detector re-enables the frequency acquisition loop. Notably, a single voltage-controlled oscillator (VCO) generating the CDR clock is shared between the two loops. Since the clock frequency generated by the VCO is proportional to the control voltage of the VCO, the phase of the clock is adjusted by changing the control voltage of the VCO for a short period of time, whereas the control voltage is changed permanently for the frequency acquisition. In the front-end, a limiting amplifier, which consists of five-stage cascaded gain stages, restores the low-swing signal from TIA to the logic level signal because this CDR uses a linear phase detection scheme that requires logical processing of data before recovery. The linear phase detection scheme provides predictable loop dynamics of the CDR as compared to the non-linear phase detection, which is represented by bang-bang phase detection or Alexander phase detection. However, a large amount of power is dissipated in this CDR structure owing to the limiting amplifier stage and the logic gate operating at a high data rate. Further detailed explanations and comparisons of the phase detecting schemes can be found in many publications, such as [104]. With the implementation of the CDR, the receiver achieves an exceptional sensitivity of −19.5 dBm at the data rate of 20 Gb/s. In [44], the fabrication of an optical TX in 0.13-µm technology is also provided, and the overall power consumption of the transmitter and the receiver is 1.25 W per channel. In [77,124], a National Taiwan University group achieved a data rate of 25 Gb/s per channel using a similar structure. In order to overcome the limited speed of the conventional linear phase detector in [44], the studies in [77,124] replaced the conventional linear phase detector with a mixer-based phase detector. While fabricated in 65-nm CMOS technology, the front-end circuit and the CDR circuit dissipate 40 mW and 100 mW, respectively, at 25 Gb/s per channel.

**Figure 44:**
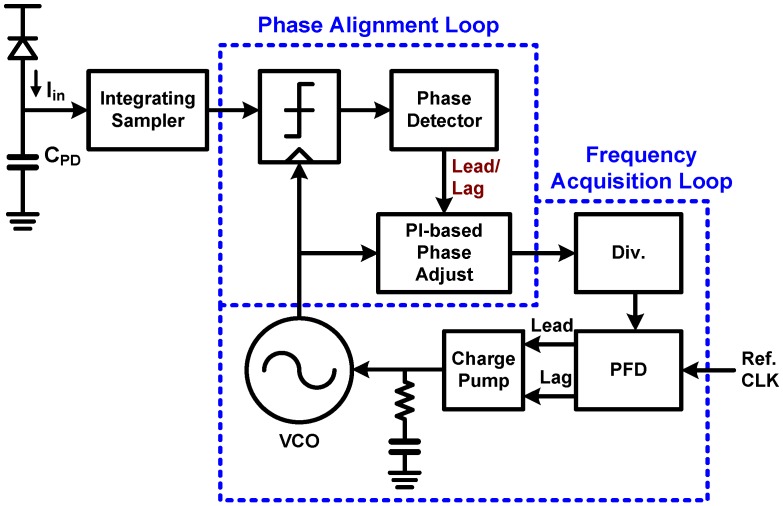
Dual loop CDR with integration-based front-end presented in [78].

The subsequent CDR example to be introduced was [93] presented by a Stanford group in 2008. The work in [93] adopted an integration-based front-end presented in [125], as shown in Figure 44. Unlike the sequential locking CDR presented in [44], the work in [93] adopted a dual-loop CDR architecture where the phase alignment and frequency acquisition loops operate simultaneously. In this type of CDR, the loop bandwidth of a loop should be set an order of magnitude lower than that of the other loop, in order to prevent the interference between the loops [119,126]. In [93], the loop bandwidth of the phase alignment loop was designed to be lower than that of the frequency loop to suppress the jitter transfer caused by the input data. Contrary to the sequential locking CDR, the VCO is not shared between the loops. An additional phase adjusting circuit based on a phase interpolator (PI) is used in the phase alignment loop. It cannot generate a clock but can change the phase of the input clock provided by the VCO according to the relative phase information extracted from the non-linear phase detector. The phase-adjusted clock is fed back into the VCO through a phase-frequency detector (PFD) so that the output phase of the VCO is eventually aligned with the data. Fabricated in 90-nm CMOS technology, the integration-based front-end and the CDR circuit consume 23 mW and 35 mW, respectively, and achieve receiver sensitivity of −5.4 dBm at a data rate of 16 Gb/s.

**Figure 45:**
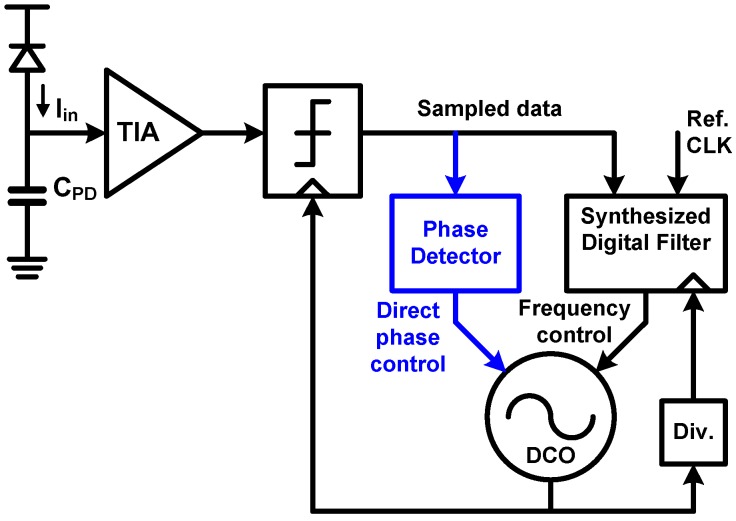
All-digital CDR presented in [74].

As CMOS technology scales down, many non-idealities appear in analog circuit components because the scaling mainly focuses on digital circuit elements. In order to overcome the various performance degradations introduced by the analog loop filters in previous CDR examples in [44,77,93,124], a recently developed all-digital CDR technology, which replaces the analog loop filter with a digitally synthesized logic, was adopted in an optical receiver by the authors’ group in [89]. In deep-submicron CMOS technology, a leakage current flowing through a MOS capacitor becomes no longer negligible due to the direct tunneling across the gate oxide [127]. Therefore, a considerable amount of current flows through the loop filter capacitor, leading to a static phase error and a deterministic jitter [128,129,130,131]. Moreover, the performance of charge-pumps is degraded owing to the reduced voltage headroom and reduced output impedance of the CMOS devices, which also results in static phase error and jitter. In order to eliminate the analog components, the VCO and analog loop filter are replaced by a digitally-controlled oscillator (DCO) and a synthesized digital logic, respectively, in [89]. While the input analog voltage of a VCO adjusts the output clock frequency, the DCO frequency is determined by a multi-bit digital code. The digital code is achieved by processing the sampled data and reference clock using a digital loop filter. On the other hand, the main drawback of this all-digital approach is the long loop latency, which introduces a phase dithering and loop stability issue. Because of the relatively low operating speed of digital logic, a frequency-divided DCO clock is used for operating the digital filter. Therefore, the loop latency introduced by the digital filter becomes tens of multiples of the bit period, since the latency of the digital filter is a few multiples of the digital clock period. In [89], a direct path without the filtering process, which provides only a phase adjustment, is separated from the main loop. As a result, fast phase response is maintained while the drawbacks of analog loop filter are overcome. The block diagram of the receiver presented in [89] is shown in Figure 45. The receiver fabricated in 65-nm CMOS achieves a data rate of 26.5 Gb/s while consuming 254 mW. Notably, the all-digital approach becomes more effective as CMOS technology continues to scale down. 

## 7. Summary and Outlook

This paper presents the history and review of the CMOS IC technology for high-speed photo-detection without relying on complex expressions or formulas, in order to provide an insight on the technology even for the readers who are not professional circuit designers. Recent high-speed photo-detecting sensor technology provides one of the most powerful forces which drive the evolution of optical links into the short-reach applications. On the other hand, another remarkable breakthrough has come from the CMOS IC technologies which convert the current-mode signal from the PD into the VLSI-compatible voltage-mode signal, because the CMOS platform provides low-power consumption, low cost, and compatibility with high-density CMOS logic blocks, which are highly required for the short-reach optical links. Because the CMOS IC for photo-detection bridges the two historic technologies of the fiber optics and the silicon VLSI systems, there is a high demand for a guidebook which introduces the technology to non-experts on IC, including optical device engineers. However, to the best of the authors’ knowledge, there has been no publication which offers good overview and summary of the CMOS IC technologies for high-speed photo-detection. The technology becomes more and more complex as the history deepens, and therefore it becomes more difficult for beginners to catch up with, as there is no publication that provides an overview of the technology evolution. For that purpose, in this paper, tens of technical papers which present high-speed photo-detecting circuits with strict verifications of theory based on IC fabrication results are thoroughly selected, reviewed, and categorized. In addition, several papers which provide a technical background of such IC design are introduced as well.

Looking ahead, the present electrical link will be ‘eventually’ replaced by the optical link, because of inherent bandwidth limit of electrical channel. The matters are when and which speed, but the ending will not change, although there are many impressive efforts to extend the limit of electrical link. The authors also believe that the platform of the optical link will be CMOS technology and thus CMOS interface circuit will play an important role. Based on the trend of last 20 years, which is described in this paper, the future optical receiver is expected to include the following features:

1. Circuit and system topologies which utilize the advantages of highly-scaled CMOS technology, such as the inverter-based TIA and all-digital CDR. It is because advanced CMOS technology focuses on optimizing digital circuits.
2. Circuit techniques to overcome the noise-bandwidth tradeoff in front-end circuit, such as the inductive peaking techniques, the equalization scheme, and the integration-based circuit. Since it is obvious that the front-end circuit has been and will be the bottleneck, both in terms of speed and SNR, it is highly required to break the tradeoff.

## Figures and Tables

**Table 1:** Summary of monolithic and hybrid-integrated optical transceivers.

	[44]	[45]	[47]	[50]	[52]	[55]	[56]	[43]
**Integration method**	Monolithic	Monolithic	Monolithic	Monolithic	Hybrid	Hybrid	Hybrid	Hybrid
**Technology (EIC)**	130 nm SOI	130 nm SOI	130 nm SOI	180 nm Bulk	90 nm bulk	65 nm bulk	28 nm bulk	40 nm bulk
**Technology (PIC)**					GaAs	GaAs	28 nm SOI	130 nm SOI
**Wavelength**	1535–1555 nm	1549–1554 nm	1560 nm	1280–1295 nm	850 nm	850 nm	1556 nm	1550 nm
**# of WDM channels**	N/A	4	N/A	9	N/A	N/A	N/A	4
**Max. data rate/CH**	10 Gb/s	10 Gb/s	25 Gb/s	5 Gb/s	25 Gb/s	10 Gb/s	25 Gb/s	20 Gb/s
**Total throughput**	20 Gb/s	40 Gb/s	25 Gb/s	45 Gb/s	25 Gb/s	600 Gb/s	25 Gb/s	80 Gb/s
**TX**								
**Modulation type**	MZI	MZI	MRR	MRR	VCSEL	VCSEL	MRR	MRR
**ER**	5–6 dB	>4 dB	6.9 dB	6.9 dB	5.1 dB	5.6 dB	6.5 dB	>7 dB
**Power/CH**	N/A	575 mW	208 mW	N/A	46 mW	69.5 mW	72.5 mW	32.3 mW
**Energy efficiency/CH**	N/A	57.5 pJ/b	8.32 pJ/b	N/A	1.84 pJ/b	6.95 pJ/b	2.9 dB	1.6 pJ/b
**RX**								
**PD type**	N/A (external)	N/A (external)	Ge waveguide	Si (defect) waveguide	GaAs (top illumination)	N/A	N/A	Ge waveguide
**Sensitivity (BER of 10^−12^) @ max. data rate**	−19.5 dBm	−15 dBm	−6 dBm	−7.5 dBm	−6 dBm @ 22 Gb/s	−16 dBm (estimated)	−8 dBm	−7.2 dBm
**Power/CH**	N/A	120 mW	48 mW	N/A	44.4 mW	68.2 mW	50 mW	11.6 mW
**Energy efficiency/CH**	N/A	12 pJ/b	1.92 pJ/b	N/A	1.78 pJ/b	6.82 pJ/b	2 pJ/b	0.73 pJ/b
**Total power**	2.5 W	3.5 W	256 mW	675 mW	90.4 mW	8.26 W	122.5 mW	175.6 mW
**Total energy efficiency**	125 pJ/b	87.5 pJ/b	10.2 pJ/b	15 pJ/b	3.62 pJ/b	13.77 pJ/b	4.9 pJ/b	2.2 pJ/b
